# Selection shapes the landscape of functional variation in wild house mice

**DOI:** 10.1186/s12915-021-01165-3

**Published:** 2021-11-19

**Authors:** Raman Akinyanju Lawal, Uma P. Arora, Beth L. Dumont

**Affiliations:** 1 The Jackson Laboratory, 600 Main Street, Bar Harbor, Maine 04609 USA; 2 Tufts University, Graduate School of Biomedical Sciences, 136 Harrison Ave, Boston, MA 02111 USA

**Keywords:** Genetic diversity, *Mus musculus*, Commensalism, Genetic disorder, Mendelian disease, Adaptation, Positive selection, Evolution, Amylase, Metabolism

## Abstract

**Background:**

Through human-aided dispersal over the last ~ 10,000 years, house mice (*Mus musculus*) have recently colonized diverse habitats across the globe, promoting the emergence of new traits that confer adaptive advantages in distinct environments. Despite their status as the premier mammalian model system, the impact of this demographic and selective history on the global patterning of disease-relevant trait variation in wild mouse populations is poorly understood.

**Results:**

Here, we leveraged 154 whole-genome sequences from diverse wild house mouse populations to survey the geographic organization of functional variation and systematically identify signals of positive selection. We show that a significant proportion of wild mouse variation is private to single populations, including numerous predicted functional alleles. In addition, we report strong signals of positive selection at many genes associated with both complex and Mendelian diseases in humans. Notably, we detect a significant excess of selection signals at disease-associated genes relative to null expectations, pointing to the important role of adaptation in shaping the landscape of functional variation in wild mouse populations. We also uncover strong signals of selection at multiple genes involved in starch digestion, including *Mgam* and *Amy1*. We speculate that the successful emergence of the human-mouse commensalism may have been facilitated, in part, by dietary adaptations at these loci. Finally, our work uncovers multiple cryptic structural variants that manifest as putative signals of positive selection, highlighting an important and under-appreciated source of false-positive signals in genome-wide selection scans.

**Conclusions:**

Overall, our findings highlight the role of adaptation in shaping wild mouse genetic variation at human disease-associated genes. Our work also highlights the biomedical relevance of wild mouse genetic diversity and underscores the potential for targeted sampling of mice from specific populations as a strategy for developing effective new mouse models of both rare and common human diseases.

**Supplementary Information:**

The online version contains supplementary material available at 10.1186/s12915-021-01165-3.

## Background

House mice (*Mus musculus*) are the premier mammalian model system for biomedical research. However, as a consequence of their unique origins from a small pool of founder animals [1], classical inbred mouse strains capture a limited subset of the genetic variation found in wild mouse populations [2, 3]. Indeed, inbred mice form a monophyletic group within *Mus musculus* [2]. Additionally, at > 97% of genomic loci, genetic variation across inbred mice can be reconciled into fewer than ten distinct haplotypes [1]. Thus, inbred mouse genomes harbor numerous “blindspots” over which there is limited genetic diversity that can be linked to phenotypic variation. Furthermore, due to their history of selective breeding for traits of interest and outcrossing between divergent house mouse subspecies, the complex multiallelic nature of trait variation in current panels of inbred strains may not faithfully model complex trait architecture in natural populations, including humans [4].

Wild house mouse genomes represent a largely unexplored reservoir of potential disease-associated genetic variation. Several lines of evidence serve to powerfully illustrate this unrealized potential. First, wild-derived inbred mice, which capture natural variation in a fixed, inbred state, are commonly outliers in strain surveys of disease-related phenotypes [5]. Second, a recent exome sequence analysis of a panel of 26 wild-derived inbred strains identified 18,496 non-synonymous variants that are not segregating among common classical inbred strains [2]. Although the phenotypic effects of these variants are not known, many are undoubtedly functional. Finally, phenotypic surveys of wild-caught house mice have already uncovered significant variation in multiple disease-associated traits, including body mass, metabolism, and behavior [6, 7].

Although wild mice harbor increased genetic variation relative to the classical inbred strains, the population genomic organization and global distribution of wild mouse diversity remain largely unknown. In humans, a significant body of genetics research has underscored the role of adaptation in shaping global patterns of diversity, including variants linked to disease risk and incidence [8]. For example, alleles that conferred a survival advantage to ancient humans during times of starvation have been linked to metabolic disorders in contemporary, food-secure modern human societies [9]. The evolution of malaria resistance has also led to high rates of sickle cell anemia in certain human populations [8, 10]. Similarly, many genes associated with the adaptive evolution of the human brain are linked to neuropsychiatric and neurodevelopmental diseases, including autism and schizophrenia [11–15]. In contrast, the extent to which natural selection may have shaped genetic diversity and disease susceptibility in wild house mice has not been thoroughly explored.

House mice are a species complex composed of three principle subspecies that diverged from a common ancestral population on the Indian subcontinent ~ 500,000 years ago [16]. *Mus musculus castaneus* is endemic to Southeast Asia. The native range of *M. m. musculus* extends from Eastern Europe to Northern Asia. *M. m. domesticus* is native to the Middle East and Western Europe. Approximately 10,000 years ago, *M. musculus* developed a commensalism with human agricultural societies. This ecological transition was likely accompanied by dietary shifts, changes in environmental pathogens, and the emergence of new behaviors. Through human-aided dispersal over the last ~ 10,000 years, *M. musculus* have expanded their home range to Africa, Australia, and the Americas. This incredible and recent geographic expansion required further local adaptation to multiple distinct ecosystems, including arid, high-altitude, cold, and extreme heat environments, as well as exposure to new pathogens. Adaptation to these new environmental pressures has potentially left unique and detectable footprints in patterns of genomic diversity across contemporary wild mouse populations.

To evaluate the impact of local adaptation and population history on the global patterning of putatively functional wild mouse genetic variation, we analyze a set of 154 publicly available diverse wild house mouse genome sequences in an evolutionary framework. We profile the global organization of predicted functional variants across multiple populations from each of the three core house mouse subspecies and perform genome-wide scans for positive selection to assess the role of adaptation in shaping the organization of genetic diversity across populations. Overall, our study reveals the landscape of functional variation in wild house mouse populations and underscores the promise of targeted sampling of mice from specific populations and environments as a strategy for developing new models of both rare and common human diseases.

## Results

### Wild house mice capture significant, and potentially functional, diversity that is absent from inbred laboratory mice

**Fig. 1:**
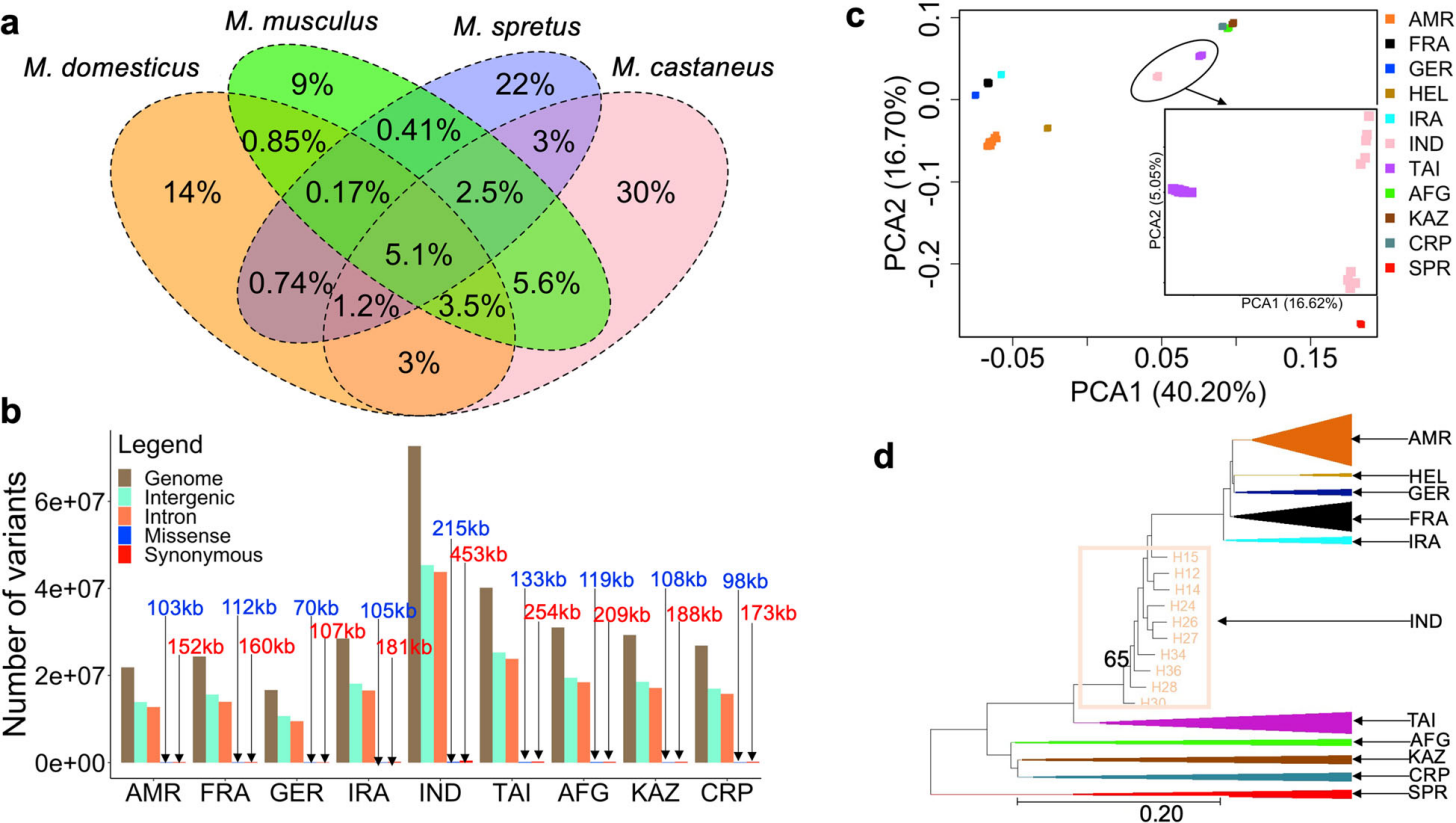
Functional annotation of wild mouse genetic diversity. **a** Venn diagram of shared and private autosomal SNPs (%) in each house mouse subspecies and species. Percentages are calculated from all ascertained variants in these samples. **b** Total numbers of autosomal variants (genome), intergenic, intron, missense, and synonymous SNPs in each *M. musculus* population. Total number of missense and synonymous variants identified in each population are annotated on their respective bar plots (kb - kilobase). **c** Principal component analysis for all 154 wild mouse genomes. The inset zooms into the two *M. m. castaneus* populations and reveals greater diversity among samples from the Indian population than the population from Taiwan. **d** Maximum likelihood phylogenetic tree from all 154 wild mouse genomes. For ease of visualization, samples from most populations are collapsed, with triangle width scaled by the number of samples in that population. One node with < 100% bootstrap support is labeled. All other nodes are supported by 100% of bootstrap replicates. The population labels are America (AMR), France (FRA), Germany (GER), Heligoland (HEL), Iran (IRA), India (IND), Taiwan (TAI), Afghanistan (AFG), Kazakhstan (KAZ), and Czech Republic (CRP), *M. spretus* (SPR)

We utilized 154 publicly available wild mouse whole-genome sequences for this study [6, 17, 18]. This panel features genome sequences from *M. spretus* (Spretus) and multiple populations from each of the three principle *M. musculus* subspecies: *M. m. domesticus* (4 populations: Eastern United States (America), France, Germany (including samples from Heligoland, a small island archipelago in the North Sea off the coast of Germany), Iran), *M. m. castaneus* (2 populations: India, Taiwan), and *M. m. musculus* (3 populations: Afghanistan (Afghan), Kazakhstan (Kazakhstani), Czech Republic (Czech)). The combined *Mus* dataset yields ~ 154 million biallelic autosomal single nucleotide polymorphisms (SNPs), including 617,156 missense, 7615 nonsense, and 985,873 synonymous SNPs. Of these, 15,104 SNPs in 6788 unique genes are predicted to be highly deleterious and disrupt gene function. Within *M. musculus* (*n* = 146 genomes), there are ~ 121 million autosomal SNPs, including 772,614 synonymous, 493,090 missense, 6216 nonsense, and 12,396 highly deleterious SNPs. Consistent with prior work [19], we observed the highest genome-wide nucleotide diversity in *M. m. castaneus* (0.0249), followed by *M. m domesticus* (0.0172), and *M. m. musculus* (0.0160). Variant statistics for each population and subspecies are provided in Figs. 1a, b.

Approximately 69.3% of the autosomal variants in *M. m. domesticus*, 63.9% of *M. m. castaneus* autosomal variants, and 53.7% of *M. m. musculus* autosomal variants are not segregating in panels of common inbred mouse strains*.* Within *M. musculus,* 13,023 of the variants found only in wild mice are predicted to be highly deleterious. Although a subset of these variants may be false positives, it is nonetheless evident that wild house mouse genomes harbor substantial unexplored and potentially functional genetic variation.

### Patterns of genetic relatedness among wild mouse samples

As our dataset was compiled from multiple prior studies [6, 17, 18], we next examined kinship and relatedness metrics among samples from each population to identify any close relatives. Fourteen pairs of animals have kinship coefficients > 0.08, indicating first- or second-degree relatedness (Additional file 1: Table S1). Importantly, we obtained qualitatively identical findings regardless of whether closely related individuals are included or excluded from our analyses (Additional file 2: Figure S1). Given the small sample sizes for several of the wild mouse populations and the robustness of our findings to the relatedness among samples, we opt to include all samples in the analyses presented below.

We then performed phylogenetic and principal component analyses (PCA) to assess genetic relationships among populations. As expected, populations from the same subspecies group together in both PCA and phylogenetic analyses (Figs. 1c, d). We utilized two independently sampled populations from the Massif Central region in France. There is no clear evidence for genetic stratification of these samples (Figs. 1c, d), and we combine these two independent population samples in our analyses. We observe greater differentiation between *M. m. castaneus* populations from India and Taiwan than between populations within other subspecies. This result is expected given the presumed ancestral origins of house mice on the Indian subcontinent and the large effective population size of this population [20], in contrast with the recent colonization of Taiwan (Fig. 1c inset). Further, consistent with these differences in population history [18, 21], genome-wide heterozygosity is markedly reduced in the Taiwanese population compared to the Indian population (4% vs 25%). The American population and German samples from Heligoland of *M. m. domesticus* are differentiated from those of mainland Europe and Iran (Figs. 1c, d), underscoring the genetic impact of founder effects during the recent colonization of these geographic regions.

### Predicted functional properties of population-private variants

As a by-product of their unique demographic origins and history of local adaptation from new or low-frequency mutations, individual house mouse populations are expected to harbor unique suites of private variants, including alleles with effects on fitness. To understand the prevalence and functional impact of such alleles, we identified variants private to each population, limiting our attention to those with a minimum allele count of 2 in the focal population to alleviate the influence of sequencing and genotyping errors. Because of the small sample sizes for each population, we acknowledge the likelihood that many of the variants marked as “private” are potentially present at low frequency in other populations.

Overall, we identified ~ 31.7 million population-private autosomal variants, representing approximately 20.6% of all segregating autosomal variants in *M. musculus*. Thus, there is considerable geographic structuring of global mouse genomic diversity. Despite the prominent role of human-facilitated migration and colonization in recent house mouse history [22], individual populations continue to harbor large loads of private variants. As expected and based on the estimates of effective population sizes and recent demographic histories [20], we find the highest numbers of population private variants in the *M. m. castaneus* populations and the Iranian *M. m. domesticus* population.

Although most population private variants are in intergenic regions and are likely neutral, an appreciable fraction resides in coding regions where they may exert effects on individual fitness (Table 1). Specifically, we identified 1483 predicted loss-of-function (LOF) variants in 1205 unique genes across the nine surveyed *M. musculus* populations. Of special note, we find a private stop-gain mutation at codon position 72 of *Mdm4* (chr1:133,011,141) that is at ~ 42% frequency in the Afghan population. *Mdm4* is a negative regulator of *p53* and is upregulated in several human cancers [23, 24]. Mouse *Mdm4* homozygous knockouts are associated with embryonic lethality, decreased cellular proliferation, and neuronal developmental defects [25]. As expected given the severity of these phenotypes, we find only heterozygous carriers for the predicted loss-of-function mutant allele in wild-caught mice from the Afghan population. Similarly, in the German population, a private mutation in *Mutyh* (chr4:116815563; ~ 14% frequency) disrupts a splice acceptor site and is predicted to abolish gene function. *Mutyh* is involved in oxidative DNA damage repair and mutations in this gene are associated with hereditary forms of colorectal cancer [26] and biases in the spectra of both germline [27, 28] and somatic mutations [29]. In mice, single knockouts of *Mutyh* are not associated with observable increases in tumor incidence, but double knockouts of *Mutyh* and *Ogg1*, a base excision repair gene, exhibit increased rates of tumor formation and shortened lifespans [30]. There are currently multiple knockout and/or targeted mutation mouse models available from commercial vendors for each *Mdm4* and *Mutyh* [31]. Our analyses reveal that organic evolutionary processes have already generated natural loss-of-function alleles for these, and presumably many other, important disease-related genes.

**Table 1:** Number of coding and predicted functional variants per population

Populations	Number of private variants	Number of synonymous private variants	Number of missense private variants	Number of stop private variants	Number of predicted deleterious variants	Number of predicted LOF variants
America	1,025,466	8675	9637	155	242	151
France	2,208,483	10,190	11,521	221	350	218
Germany	545,881	2178	2506	51	74	39
Iran	3,333,440	15,430	11,548	149	235	145
India	16,025,598	70,201	35,493	447	745	359
Taiwan	3,917,054	19,969	15,957	244	410	225
Afghanistan	1,472,430	7657	6566	124	170	102
Czech Republic	1,315,866	6582	6489	112	169	106
Kazakhstan	1,872,782	9403	8902	174	230	138
Total	31,717,000	150,285	108,619	1677	2625	1483

### Detecting signals of positive selection in wild mouse genomes

Just as observed in human populations [8], local adaptation has almost certainly molded the geographic distribution of disease-associated trait variation in wild mice. To directly investigate this possibility, we carried out genome-wide scans for positive selection in each of the nine surveyed wild mouse populations.

Strong positive selection on an adaptive allele will result in its rapid sweep to high frequency or fixation in a population. This process will yield a localized reduction in genetic diversity at the selected site, a signature referred to as a “selective sweep.” The strength of this trademark signal is governed by a complex interplay of population genetic variables, including the magnitude of selection, the initial frequency of the selected allele, and the local rate of recombination.

A key challenge for the interpretation of genome-wide scans for selection is to distinguish regions truly evolving via positive selection from outliers of the neutral diversity distribution. For example, certain demographic scenarios can induce genome-wide reductions in diversity that may masquerade as pervasive positive selection [32]. One powerful approach to circumvent this challenge is to apply coalescent simulations that realistically model the ancestry of the analyzed sample to derive an empirical distribution of the test statistic under the assumption of neutrality. We estimated population-specific demographic parameters and applied coalescent simulations to approximate the neutral distribution of three population genetic diversity summary statistics in each population: *H*_p_ (pool heterozygosity) [33], *π* (nucleotide diversity) [34], and Tajima’s D [35] (see the “Methods” section). Statistics were computed in 20 kb sliding windows (10 kb step size) across the genome. This window size is less than the expected scale of linkage disequilibrium decay in previously surveyed wild mouse populations [3]. Comparing the observed and simulated distributions of each diversity statistic allowed us to define population-specific empirical cut-offs for identifying loci evolving via positive selection (Additional file 3: Figure S2). We focus on regions detected as outliers by the *H*p statistic and by at least one of the other two statistics. Additional files 4, 5 and 6: Figures S3–S5 display the genome-wide distributions of these three summary statistics in each population.

Overall, we identified 280 putative sweep regions across the four *M. m. domesticus* populations, including 18 in the American population, 145 in the French population, 132 in the German population, and 8 in the Iranian population. A total of 272 selective sweep loci were identified in *M. m. castaneus*, including 15 in the Indian population and 258 in the Taiwanese population. We uncovered 532 putative selective sweep loci in *M. m. musculus.* Of these, 58 were observed in the population from Afghanistan, 47 in the Kazakhstani population, and 434 in the Czech population. We also identified 101 candidate selective sweeps in *M. spretus.* Additional files 7, 8, 9 and 10: Tables S2–S5 present comprehensive catalogs of these candidate regions, including shared signals of positive selection between populations.

Positive selection is expected to operate exclusively on functional genomic regions, but there is no *a priori* expectation that neutrally evolving loci should be enriched for functional annotations [36]. Approximately 98.9% of the selective sweep loci reported in our analysis span at least one protein-coding gene. In contrast, in 1000 independent simulations of random size-matched genomic intervals, at most 67.8% overlapped protein-coding genes *(p* < 0.001)*.* The marked enrichment for protein-coding annotations in our selective sweep windows suggests that our candidate regions are strongly enriched for bonafide targets of positive selection.

### Cryptic structural variation manifests as false-positive signals of selection

We noted that many candidate selective sweep regions overlapped annotated segmental duplications and polymorphic structural variants previously described in laboratory mouse strains. For instance, in the Indian population of *M. m. castaneus,* we observed a sharp decrease in *H*p, *π*, and Tajima’s D at chr4:112.23–112.61 Mb, a locus spanning a cluster of paralogs in the *Skint* gene family (Additional file 11: Figure S6a). Relative to the C57BL/6 J mouse reference genome, at least 13 inbred strains carry a deletion spanning three paralogs in this region (*Skint3*, *Skint4*, and *Skint9*) [37, 38]. We analyzed patterns of read depth at the *Skint* locus in our wild mouse samples and confirmed that a single deletion allele segregates at frequencies 57%, 80%, and 82% in wild *M. m. domesticus*, *M. m. castaneus,* and *M. m. musculus* populations, respectively. The deletion frequency was 90% in the Indian population (Additional file 11: Figure S6b and S6c). These findings raise the possibility that cryptic deletions or other structural variants may commonly lead to local reductions in the number of surveyed haplotypes, and as expected, concomitant loss of diversity. Critically, prior studies demonstrate that wild house mouse populations harbor high loads of structural variation [17, 39] which, if ignored, could yield abundant false-positive signals of positive selection.

We applied a post-hoc read depth filter to mask regions of the genome present in a non-diploid state (see the “Methods” section). After applying this key quality control step, the number of putative selection regions decreased from 1180 to 1084. Thus, approximately 8% of all regions originally identified in our analysis are likely false-positive signals attributable to structural variation. Our findings underscore the significant impact of cryptic structural variation on the genome-wide inference of positive selection and emphasize the importance of masking copy number variable regions in QC processing for genome-wide scans (e.g., [40]). All analyses presented below focus on this refined set of candidate positive selection regions.

### Functional classification and annotation of putative selection regions

**Fig. 2:**
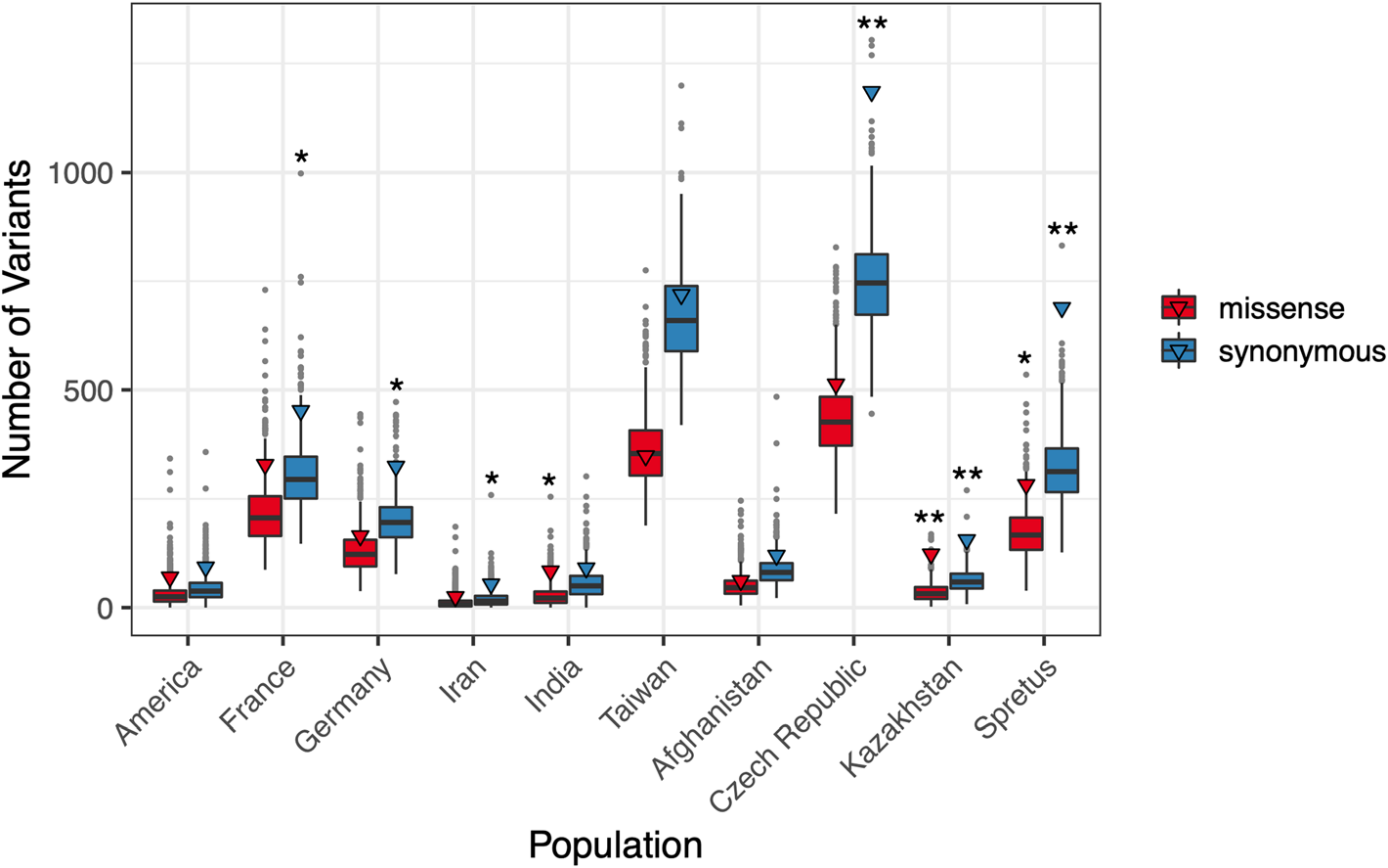
Functional classification of variants found within selective sweep windows. Boxplots display the distribution of the number of missense (red) and synonymous (blue) variants in 1000 sets of randomly sampled windows size-matched to the number of positive selection regions identified in each population. Outliers are designated by gray points. The observed number of missense and synonymous variants in candidate positive selection regions are designated by a triangle. *P < 0.05; **P < 0.01

We sought to probe the functional impact of the putative positive selection signals documented in each population. First, we asked whether selection windows are enriched for non-synonymous sites relative to genome-wide expectations. In three surveyed populations, we find evidence for a significant excess of missense variants in selection windows relative to genome-wide expectations (Fig. 2; India: *P* = 0.03, Kazakhstan: *P* = 0.009, *M. spretus*: *P* = 0.038). Six populations exhibit a significant excess of synonymous variants (Fig. 2; France: *P* = 0.405, Germany: *P* = 0.017, Iran: *P* = 0.0479, Czech Republic: *P* = 0.003, Kazakhstan: *P* = 0.002, *M. spretus*: *P* = 0.002).

**Fig. 3:**
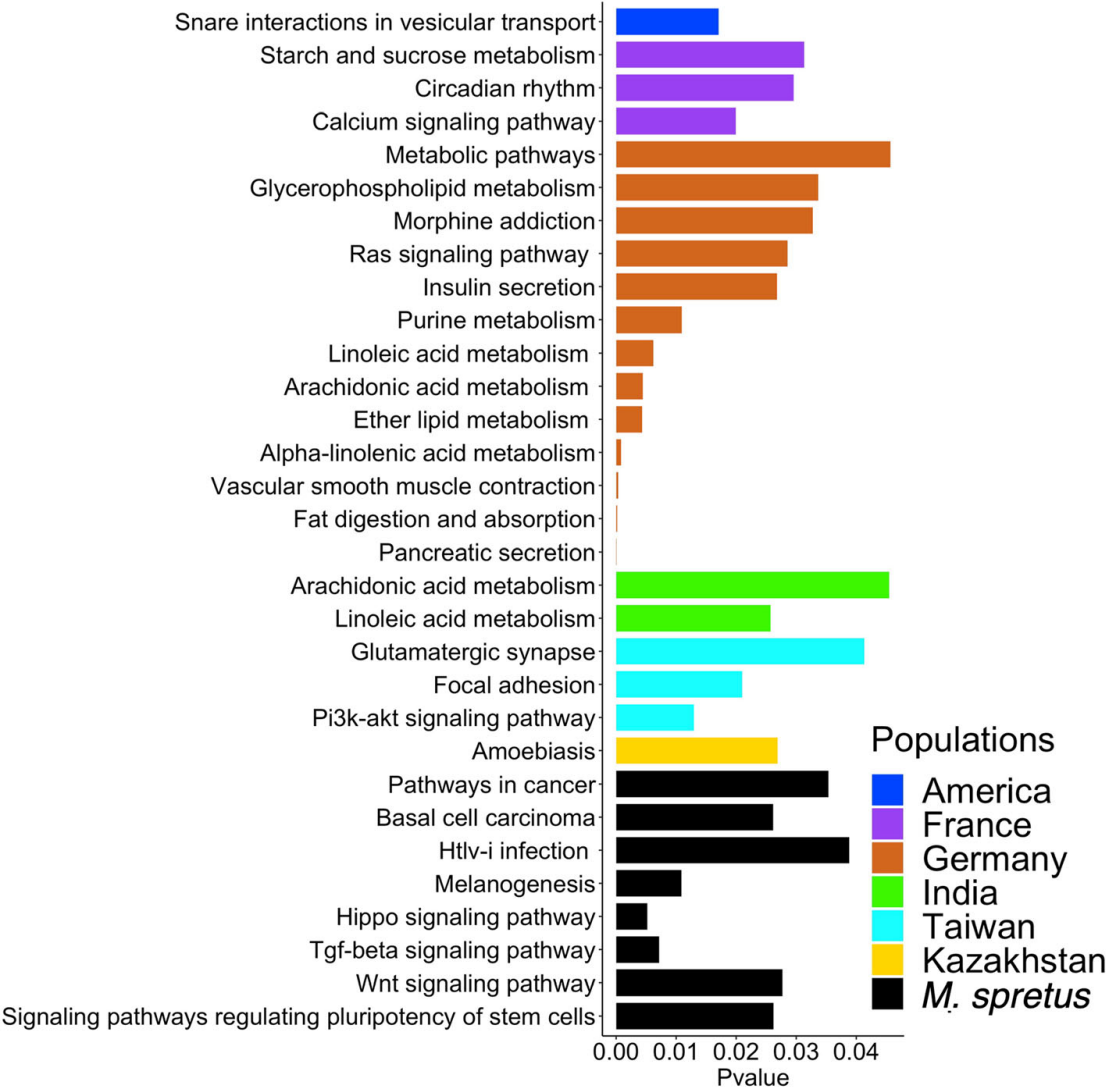
KEGG pathway analysis of genes within positive selection windows. Only populations with significant enrichment (*p* < 0.05) for each specified pathway are shown. See Additional files 12, 13, 14 and 15: Figures S7–S10 for GO analysis results in each population

Next, to understand the broad biological impact of selection across the genome, we performed Gene Ontology (GO) and Kyoto Encyclopedia of Genes and Genomes (KEGG) enrichment analyses using all *Mus musculus* gene annotations as background and selective sweep genes from each population as the foreground sets. Figure 3; Additional files 7, 8, 9 and 10: Tables S2–S5, and Additional files 12, 13, 14 and 15: Figures S7–S10 provide comprehensive summaries of findings from these functional enrichment analyses.

KEGG analysis uncovered several biologically enriched pathways associated with multiple metabolic functions (see Fig. 3). For instance, genes related to “starch and sucrose metabolism” (*Amy2a5*, *Amy1*, *Sis*) are enriched among selection targets in the French population. In the German population, genes implicated in “fat digestion and absorption” (*Pla2g2f*, *Pla2g2d*, *Pla2g2a*, *Pla2g5*, *Cel*) and “insulin secretion” (*Gnaq*, *Kcnmb2*, *Kcnn1*, *Adcy8*) are over-represented among putative selective sweep genes. In the Indian population, we find an excess of genes involved in “linoleic acid metabolism” (*Cyp2j8*, *Cyp2c38*). KEGG analysis also highlights several pathways associated with disease including “basal cell carcinoma” (*Wnt6*, *Gli2*, *Wnt10a*) and “htlv-i infection” (*Smad4*, *Wnt10a*, *Prkacb*, *Wnt6*, *Tgfbr2*) in *M. spretus*.

A GO analysis of selection signals also uncovered significant enrichment for annotations linked to diverse biological functions. For instance, in the *M. m. domesticus* American population (Additional file 12: Figure S7), we report enrichment of genes with functions in “chromatin organization” (*Cdan1*, *Zfp462*). Genes that function in “cell cycle arrest” (*Tgfb2*, *Il12b*, *Apbb2*, *Brinp3*), “response to hypoxia” (*Acvrl1*, *Tgfb2*, *Epas1*, *Cd38*, *Ece1*, *Plod1*), “rhythmic process” (*Suv39h2*, *Prkdc*, *Cry1*, *Rora*, *Csnk1d*), and “sensory perception of sound” (*Thrb*, *Strc*, *Map1a*, *Nav2*, *Ccdc50*, *Fam107b*) are over-represented among selection targets in the French population. In the German population, we report an excess of putative sweep genes with roles in “negative regulation of t-cell proliferation” (*Pla2g2f*, *Pla2g2d*, *Pla2g2a*) and “autophagy” (*Map1lc3a*, *Lrrk2*, *Mfn2*, *Trp53inp2*, *Vps39*). Genes implicated in “t-RNA binding” (*Xpo5*, *Trmt1*) are over-represented among selection signals in the Iranian population.

In the Indian *M. m. castaneus* population*,* selection windows are enriched for genes annotated to the GO term “innate immune response” (*Cr2*, *Cr1l*, *Herc6*). Within the Taiwanese *M. m. castaneus* population, genes under selection are over-represented in the biological processes “hemopoiesis” (*Lyn*, *Meis1*, *Cdk6*, *Txnrd2*, *Brca2*), “erythrocyte differentiation” (*Acin1*, *Lyn*, *Fech*, *Jak2*), and “detection of chemical stimulus involved in sensory perception of smell” (*Olfr853*, *Olfr830*, *Olfr866*, *Olfr832*, *Olfr870*, *Olfr851*, *Olfr872*, *Olfr829*, *Olfr845*, *Olfr869*) (see more at Additional file 13: Figure S8). Selection targets in *M. m. musculus* are similarly over-represented in diverse biological processes including “behavioral response to nicotine” (Afghan population; *Chrna3*, *Chrna5*); “postsynaptic membrane” (Kazakhstani population; *Grin3a*, *Grid1*, *Lrrtm4*, *Psd3*), and “regulation of cardiac muscle contraction” (Czech population; *Ryr2*, *P2rx4*, *Adora1*, *Ank2*, *Tnni3k*, *Smad7*) (Additional file 14: Figure S9). In *M. spretus*, genes evolving via positive selection are enriched for the GO terms “cellular response to hypoxia” (*Fndc1*, *Clca1*, *Mgarp*, *S100b*), “regulation of cell proliferation” (*Smad4*, *Sparc, Fanca*, *Pbx1*, *Tgfbr2*), and “kidney development” (*Pkhd1*, *Smad4*, *Fbn1*, *Gli2*) (Additional file 15: Figure S10)

### Targets of positive selection in wild house mouse populations

Our catalogs of positive selection emphasize several known and recurrent targets of adaptive evolution in mammals. Below, we highlight several of the strongest signals identified in each surveyed population.

In the American population of *M. m. domesticus*, the strongest peak (chr4: 129.62–129.64 Mb) overlaps a gene-rich locus spanning *Txlna*, *Ccdc28b*, and *Tmem234*. *Txlna* is an interleukin 14 gene expressed in various tumor cells and involved in cell proliferation of hepatocellular carcinomas [41]. *Ccdc28b* functions in ciliogenesis and is associated with Bardet–Biedl syndrome [42], a syndrome linked to vision loss, obesity, speech impairment, and intellectual disability. *Tmem234* is poorly studied. Future work is needed to pinpoint the target(s) of selection in this window.

In the French population, the strongest signal of positive selection is at chr10:85.1–85.2 Mb. This locus includes four genes: *Cry1*, *Mterf2*, *Fhl4*, *Tmem263*. *Cry1* is a core regulatory component of the circadian clock. Variants in this gene have been associated with sleep disorders and altered sleep patterns in diverse organisms [43]. *Mterf2* is involved in regulating mitochondrial mRNA and rRNA transcription [44], and *Fhl4* mutations can lead to hemophagocytic lymphohistiocytosis [45]. *Tmem263* plays a role in bone mineral deposition and is associated with autosomal recessive dwarfism in chickens [46]. We also identified a strong selection signal around *Epas1* (chr17:86.77–86.80 Mb). *Epas1* is a transcription factor that is activated under hypoxic conditions and prior studies have linked variation at this gene to high-altitude adaptation in mammals and birds [47, 48]. Intriguingly, mice from this population were collected from the mountainous Massif Central region of France [17], where oxygen levels may be reduced to 81% of their values at sea level.

In the German population of *M. m. domesticus*, the strongest peak spans *Cdan1*, *Ttbk2*, and *Stard9* on chromosome 2 (120.63–120.81 Mb). *Cdan1* functions in chromatin assembly with mutations in the gene linked to congenital dyserythropoietic anemia [49]. *Ttbk2* plays a key role in ciliogenesis, the development of the cerebellum, and balance coordination [50]. *Stard9* is involved with the regulation of spindle pole assembly and has been linked to mitotic arrest and cancer [51]. A selective sweep was also found around *Cdan1* and *Ttbk2* in the American and French populations of the same subspecies (Additional file 7: Table S2), suggesting that this locus may have been targeted by selection in multiple *M. m. domesticus* populations. To our knowledge, our report represents the first evidence for adaptive evolution at the *Cdan1*/*Ttbk2* locus, although the specific environmental pressures that have led to these sweep signals remain to be determined.

**Fig. 4:**
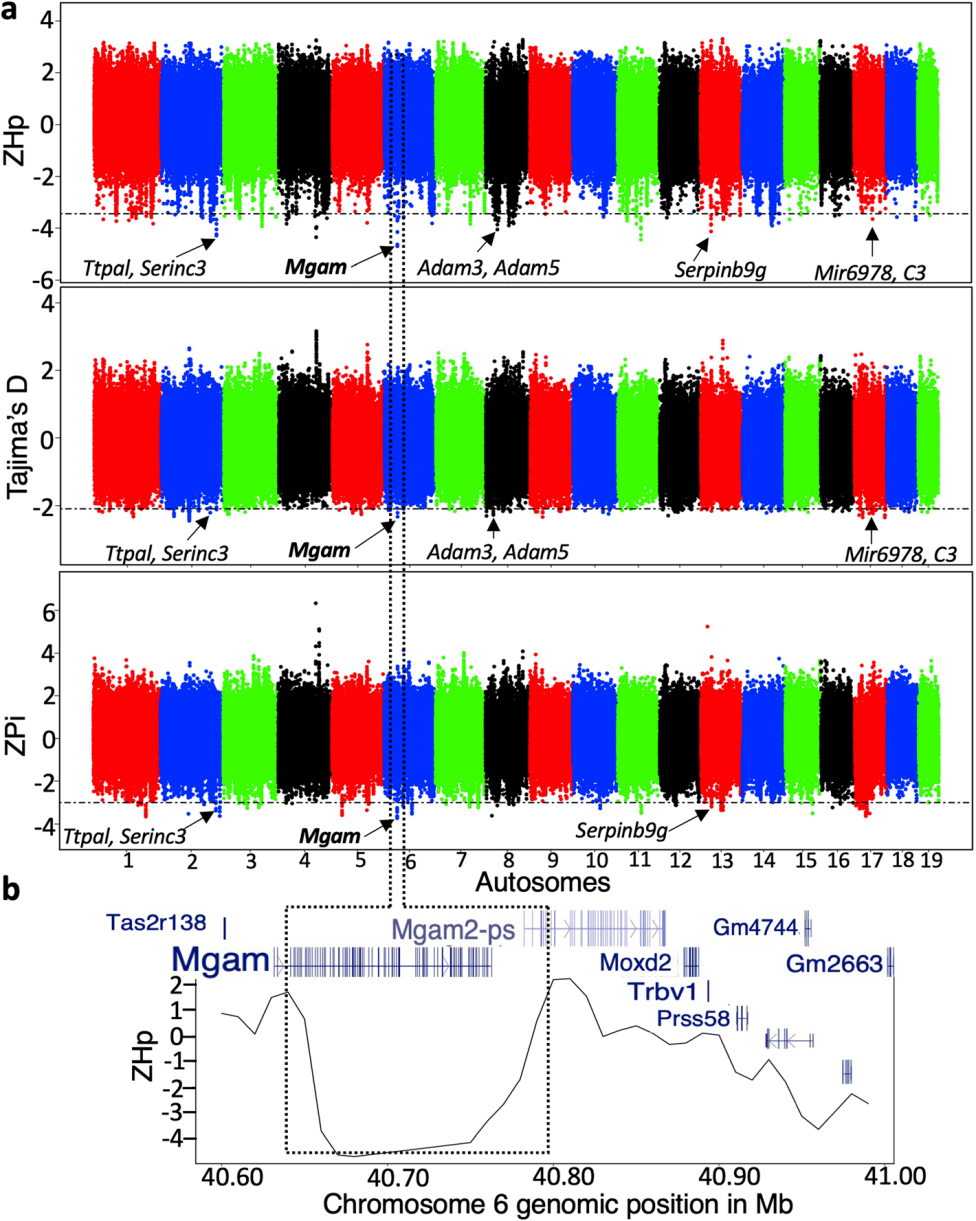
Signatures of positive selection in the Iranian population of *M. m. domesticus*. **a** The genomic distribution of normalized *H*_p_, Tajima’s *D*, and *π* (Pi). Horizontal lines on the first three panels correspond to the genome-wide significance threshold derived from neutral diversity simulations. Each dot represents a 20-kb window. **b** provides a close-up of ZH_p_ across the *Mgam* locus on chromosome 6: 40.67–40.79 Mb. A pronounced drop in ZH_p_ is specifically localized to the coding region of *Mgam*

The most notable peak in the Iranian population localizes to chr6:40.67–40.79 Mb and spans a single gene, *Mgam* (Fig. 4)*. Mgam* also exhibits signals of adaptive evolution in the Afghan population of *M. m. musculus* (Additional files 7 and 9: Tables S2 and S4)*. Mgam* encodes a starch digestion enzyme and prior work has implicated this gene in the adaptation to starch-rich diets during dog domestication [52] and the transition to agriculture in ancient Andean humans [53].

The strongest peak in the Indian population of *M. m. castaneus* spans *Zswim2* and *Fam171b* (chr2:83.87–83.93 Mb). *Zswim2* is an E3 ubiquitin-protein ligase that is involved in the regulation of apoptosis [54]. *Fam171b* is less well studied. The strongest selection signal in the Taiwanese population bridges *Ttpal*, *Serinc3*, and *Pkig* at chromosome 2:163.59–163.67 Mb. This locus is also under positive selection in the Indian, Iranian, and Kazakhstani populations. *Ttpal* is a lipid transporter*, Serinc3* functions in viral immunity [55], and *Pkig* plays a role in osteogenesis (Additional file 8: Table S3).

In both the Afghan and Czech populations of *M. m. musculus*, the most pronounced selective sweep signal encompasses *Lrp5* (chr19:3.65–3.73 Mb; Additional file 9: Table S4). *Lrp5* has diverse roles in the maintenance of bone mass, eye development, and cholesterol homeostasis [56], and has been implicated in osteoporosis [57]. In the Kazakhstani population, the strongest signal of positive selection resides on chromosome 15 (3.25–3.31 Mb) and spans *Ccdc152* and *Selenop*. This locus also exhibits a weaker signal of positive selection in the Czech population. *Ccdc152* is poorly studied. *Selenop* encodes a seleno-protein that transports selenium to the plasma, where it is functionally important in thyroid metabolism and protection against oxidative stress [58]. Another notable peak located at chr7:56.23–56.25 Mb in the Kazakhstani population spans *Herc2* and *Oca2*. Genetic variation in both *Herc2* and *Oca2* is associated with pigmentation of skin, hair, and eyes. *Oca2* plays a role in melanin synthesis and eye color determination and has been linked to albinism [59, 60]. Analyses of selection in diverse human populations have revealed parallel selection pressures at this locus [60].

The most prominent selective sweep signal in *M. spretus* is found at chr8:67.69–67.75 Mb (Additional file 10: Table S5)*.* This interval spans a single gene–*Psd3*–that has been associated with immune disease and cancer [61]. Two other prominent peaks are found at chromosomes 16 (29.58–29.65 Mb) and 14 (27.48–27.51 Mb) overlapping *Opa1* and *Ccdc66*, respectively. *Opa1* is a dynamin-like GTPase gene that functions at the inner mitochondrial membrane and plays a critical role in visual perception [62]. *Ccdc66* is implicated in retinal morphogenesis [63].

### Selective sweeps are enriched for GWAS hits and genes implicated in Mendelian diseases

We noted that many regions of positive selection in wild mouse genomes overlapped known disease-associated and disease-causal genes in humans (Additional file 16: Table S6). Across all surveyed populations, 54.3% of genes with signals of positive selection can be assigned to at least one disease-relevant phenotype in the Online Mendelian Inheritance in Man (OMIM) database. This represents a significant increase over simulation-based expectations (*p =* 0.03). Similarly, 55.4% of all candidate genes within selective sweep windows overlap at least one trait in the genome-wide association study (GWAS) catalog, again in excess of expectations from random simulations (*p =* 0.005)*.*

**Fig. 5:**
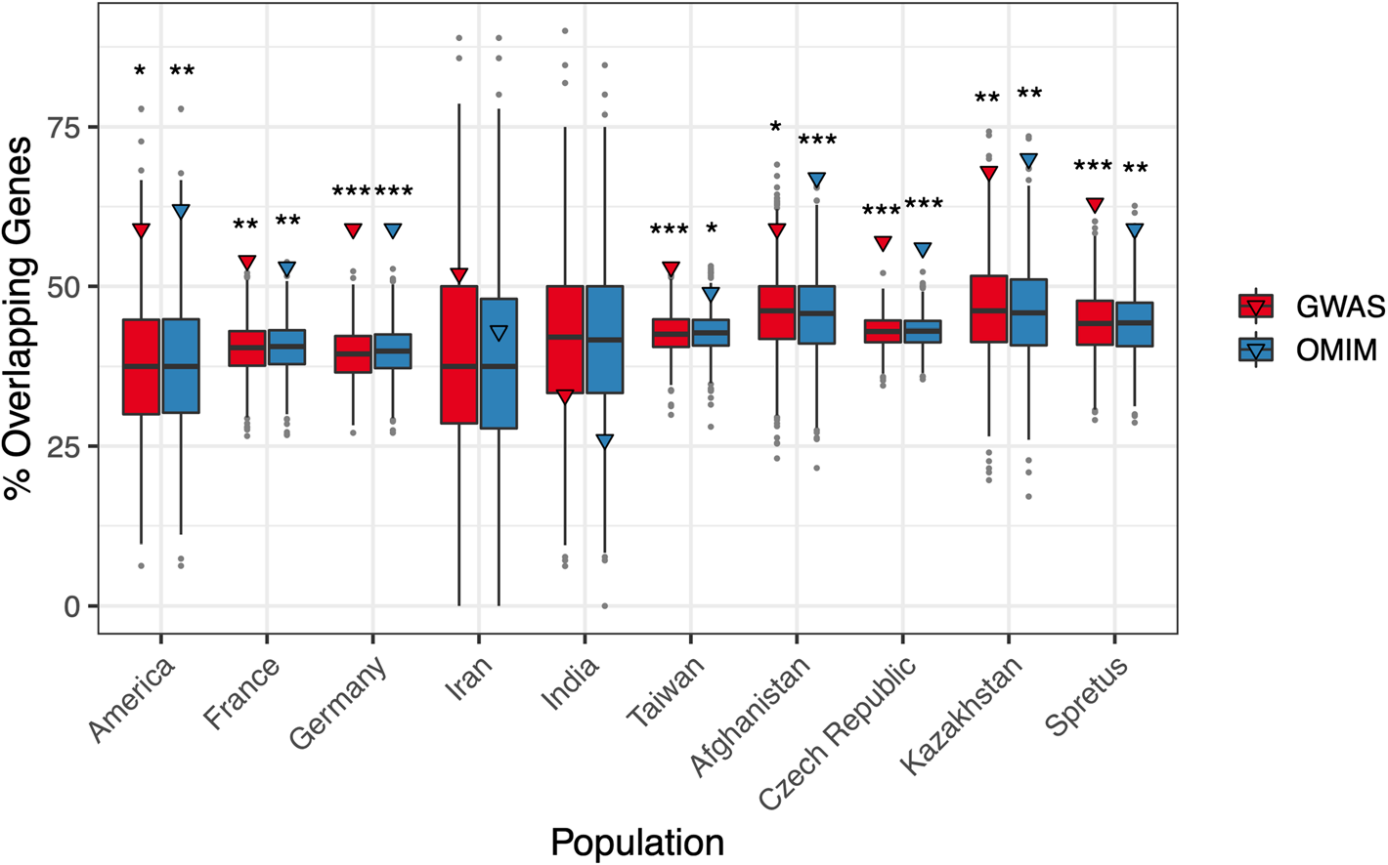
Percentage of genes in selective sweep windows that overlap genes in the OMIM database and human GWAS catalog. Boxplots display the distribution of the number of overlapping GWAS (red) and OMIM (blue) genes in 1000 sets of randomly sampled windows size-matched to the number of positive selection regions identified in each population. Outliers are designated by gray points. The observed number of positive selection genes overlapping GWAS and OMIM database hits are designated by a triangle. *P < 0.05; **P < 0.01; ***P < 0.001

To investigate these trends on a per-population basis, we estimated the fraction of sweep genes that overlap OMIM genes in each population. This quantity varies considerably across the surveyed populations, ranging from 26% in the Indian population of *M. m. castaneus* to 70% in the Kazakhstani population of *M. m. musculus* (Fig. 5). Similarly, populations vary in the proportion of sweep genes that overlap GWAS hits (33%–68%, Fig. 5). Overall, these results suggest that targets of positive selection in most of wild mouse populations are significantly enriched for disease-associated genes.

### An initial test of the effect of adaptive evolution on gene expression changes

An enduring question in evolutionary biology concerns the relative roles of adaptation on coding sequence changes versus gene expression [6, 64, 65]. We leveraged published RNA-seq data [17] from a subset of the wild *M. m. domesticus* mice used in these genome-wide selection scans to ask whether genes under positive selection show stronger patterns of differential regulation across populations than expected. Overall, we find clear evidence for differential regulation of genes under positive selection in the brain (*P* < 0.0049), heart (*P* < 0.0001), muscle (*P* < 0.0002), spleen (*P <* 0.0051), lung (*P <* 0.038), and testis (*P* < 0.01) (Additional file 7: Table S2 and Additional file 17: Figure S11). Of note, *Epas1* is under positive selection in the French population and is significantly upregulated in liver and muscle tissues of mice from France as compared to mice from the German population. However, we do not observe differential expression of this gene in the heart, as previously shown for high-altitude adapted deer mice [66]. We find no significant differences in *Mgam* expression levels in digestive tissues (gut, liver) among *M. m*. *domesticus* populations (Additional file 17: Figure S11), suggesting that positive selection at this locus may act on coding sites that alter enzymatic activity. This finding aligns with the pronounced drop in diversity (see Fig. 4b) restricted to coding portions of the gene, to the exclusion of upstream regulatory regions. *Cry1*, a highly conserved gene implicated in the maintenance of circadian rhythm, shows upregulation across multiple tissues in French mice compared to mice from Germany and Iran, consistent with the signal of adaptation at this locus which is restricted to the French population. Finally, *Amy1* is under positive selection in the French population and is upregulated in gut tissues from both the Iranian and French populations relative to mice from the German population. This finding is consistent with possible regulatory modes of adaptive evolution at this locus. In summary, our findings suggest that a subset of the signals identified in our genome-wide selection scan may be caused by variants with effects on gene expression, rather than protein-coding mutations.

## Discussion

Here, we analyzed the genomes of 154 wild-caught mice to assess the population-wide distribution of functional genetic diversity and establish the contribution of positive selection to the global patterning of disease-relevant trait variation. We show that a large fraction of wild mouse variation is specific to individual populations, including numerous predicted loss-of-function variants that could be useful in the context of disease modeling. Further, our work has synthesized a comprehensive catalog of candidate genes and genomic regions evolving via positive selection in diverse wild house mouse populations. Our surveyed populations inhabit distinct environments that differ in altitude, average temperature, aridity, and human population density. These environmental differences have created unique opportunities for population- and subspecies-specific adaptations, including the emergence of adaptive traits that may confer differences in disease susceptibility. Several exciting themes emerge from this catalog.

First, like many other animal species [67], genes involved in immunity and sensory perception are common targets of adaptive evolution in wild house mice. Across populations and subspecies, we identified multiple sweep regions spanning genes with immune-related functions (e.g., *Serinc3, Stat3*, *Cr2*, *Cr1l*, *Herc6*, *Dclre1c*, *Il12b*, *Prkdc*). The diverse suite of pathogens endemic to each population’s environment has likely imposed strong selective pressures on the immune system. We also document positive selection signals at multiple olfactory receptors (ORs). The OR repertoire is known to evolve rapidly, with notable gains and losses across the mammalian tree [68]. Interestingly, we find few shared signals of selection at ORs across wild mouse populations (Additional files 7, 8 and 9: Tables S2–S4). We speculate that positive selection has likely led to population-specific OR portfolios tuned to the detection of specific aromatic compounds in the prevailing environment.

Second, several genes that are evolving via positive selection in house mice are also targets of adaptive evolution in human populations. For example, *Epas1* has been implicated in high altitude adaptation in several human populations and we observed a genetic signature of recent selective sweep at this locus in mice from a mountainous region in France. Similarly, *Mgam* is evolving under adaptive evolution in both an Andean human population [53] and in wild mouse populations from Iran and Afghanistan. These instances of parallel evolution suggest that wild mice could serve as powerful models for dissecting the molecular basis of some adaptative traits in humans.

Third, our study uncovers loci that may have contributed to the development of successful commensalism between house mice and humans. Recent archeological evidence shows that mice emerged as commensals with humans approximately 14,500 cal. BP, coinciding with the establishment of the first sedentary hunter-gatherer settlements [22]. The earliest human-domesticated plants were grains [69], which also comprise a staple of wild mouse diets. However, commensalism was likely linked to an increased dietary reliance on grains and starch-rich foods, at the expense of seeds, fruits, insects, and other components of the wild mouse diet. This dietary shift potentially imposed strong selection to improve the efficiency of nutrient absorption from grains and starches. Indeed, we found clear evidence for recent positive selection at *Mgam*, a maltase-glucoamylase that plays a key role in the final stages of starch digestion. It is particularly noteworthy that signals of selection on this gene are limited to the mouse populations from Iran and Afghanistan, as these two populations coincide with some of the earliest human agricultural settlements [70] and overlap the presumed ancestral region of *M. musculus* [71, 72]. Strikingly, prior studies have also linked signals of positive selection at *Mgam* to the successful transition to agriculture in Andean human populations [53] and dietary shifts that accompanied the domestication of dogs [52]. We also identified a signal of selection near *Amy1* on chr3qF3 in the mouse population from France. *Amy1* is a presumed target of positive selection in human populations, with increased copy number linked to increased starch digestion capacity [73]. However, our data show that genetic adaptation at *Amy1* in French mice is likely rendered through short nucleotide variants, rather than copy number changes (Additional file 18: Figure S12), an observation consistent with finding in another human population [53].

Fourth, many selective sweeps in wild house mice have occurred at genes that have been implicated in human diseases and disorders (Additional file 16: Table S6). Indeed, we show that targets of positive selection in several wild mouse populations are significantly enriched for disease-associated genes compared to null expectations. For example, multiple mouse populations harbor signals of selection associated with autism spectrum disorder and speech-related impairment (e.g., *Cntnap2*, *Trrap*, *Herc2*, *Nlgn1*, and *Nalcn*), deafness (e.g., *Met*, *Ubr1*, *Pcdh15*, *Ccdc50*, *Dnmt1*, *Col11a1*, *Myo3a*, *Otogl*, *Ppip5k2*, *Slc26a4*), diabetes (e.g., *Retn*, *Cel*, *Hnf4a*), glaucoma (e.g., *Opa1*, *Asb10*), and intellectual disability (e.g., *Auts2*, *Trmt1*, *Slc4a4*, *Trappc9*, *Kcnk9*, *Lingo1*). Understanding the mechanisms of adaptation at these genes in wild mouse populations could provide critical insights into the evolutionary basis of these diseases in humans.

In addition to these major themes, our analysis also presents a cautionary tale regarding the importance of integrating data on local genomic copy numbers with diversity metrics used in selection scans. Notably, several regions of significantly reduced diversity that emerged in our analysis proved to be false positives due to the presence of cryptic segregating structural variants. For example, a signal consistent with the positive selection at the *Skint* gene cluster on chr4:112.08–112.60 Mb in the Indian *M. m. castaneus* population is an artifact due to a high-frequency deletion spanning this region (Additional file 11: Figure S6). This finding reinforces the critical importance of imposing quality control filters to eliminate structurally variant regions from genome-wide scans for selection (e.g., [40]).

Overall, our findings mirror conclusions for human populations, revealing that natural selection has shaped the geographic landscape of wild mouse variation in a manner that influences the distribution of likely disease-associated alleles. However, we note that our approach for identifying signals of positive selection is not designed to find signals of polygenic adaptation. In contrast to the hard selective sweep signatures reported here, wherein a single haplotype or variant is driven to high frequency within a population, signals of adaptation on polygenic traits typically yield so-called “soft sweep” signatures, marked by milder increases in allele frequency of the high-fitness haplotype [74, 75]. Powerful approaches for detecting polygenic adaptation have been developed in recent years (e.g., [76]), and future efforts would be well spent by applying these methodologies to the wild mouse populations studied here.

## Conclusions

Successful adaptation to a commensal environment set the stage for subsequent human-aided dispersal of house mice across the globe, including the colonization of new environments in recent history. As a consequence of this demographic history and subsequent local adaptation, mice from different geographic regions are genetically and phenotypically differentiated, and notably at many loci associated with traits with immediate relevance to human health and disease. Our analysis reveals that natural selection has played an important role in shaping global patterns of wild mouse diversity and spotlights key pathways and genes targeted by positive selection during recent house mouse evolutionary history. We anticipate that our catalog could help prioritize specific geographic areas for sampling wild mice to develop new natural mouse models of human disease or conduct genome-wide association studies in natural populations [7].

## Methods

### Whole-genome sequences

We analyzed a total of 154 previously published whole-genome sequences [6, 17, 18], including multiple populations from each of the three principle house mouse subspecies. In total, we surveyed four populations of *M. m. domesticus*, including 50 samples from the Eastern United States, 28 from France, nine from Germany (including three samples from Heligoland, a small island archipelago in the North Sea off the coast of Germany), and seven from Iran. We analyzed 30 *M. m. castaneus* genomes from two populations (Taiwan, *n* = 20; India, *n* = 10), and 22 *M. m. musculus* genomes from three populations (Afghanistan, *n* = 6; Czech Republic, *n* = 8; Kazakhstan, *n* = 8). The sequence dataset also includes eight *M. spretus* genomes from Spain. The distributions of average quality scores and read depth for each genome are shown in Additional file 19: Figure S13.

### Sequence alignment and variant calling

Fastq reads were mapped to the mm10 reference genome using the default parameters in BWA version 0.7.15 [77]. We followed the standard Genome Analysis Toolkit (GATK; version 3.8.0) pipeline for subsequent pre-processing before variant calling [78, 79]. Next, variant calling was performed on each sample using the “-ERC GVCF” mode in the GATK “HaplotypeCaller”. Samples were then jointly genotyped using the “GenotypeGVCFs” GATK function. The “output” from the joint genotyping was subjected to a series of hard filters using “--filterExpression “QD < 2.0 || FS > 60.0 || MQ < 40.0 || MQRankSum < -12.5 || ReadPosRankSum < -8.0.” The resulting hard filtered variants and previously ascertained mouse variants [80] were then used as training data for the “output” during the variant recalibration stage using both the “VariantRecalibrator” and “ApplyVQSR” option of GATK. For the latter, the truth sensitivity level to initiate filtration was set to its default (i.e., 99). Only biallelic variants passing the variant recalibration stage were included in downstream analyses.

### Variant annotation and statistics

We used SnpEff (version 4.3 t) for both variant annotation and the determination of the total number of variants within each functional class per sample and per population [81]. The numbers of shared and unique variants between each subspecies and between species were calculated using the “vcf-stats” and “vcf-isec” commands within VCFtools (version 0.1.16) [82]. Variant sharing between taxonomic groups was visualized using the “VennDiagram” R package (version 1.6.20) [83].

### Assessing genetic relatedness

Closely related samples were identified using KING (version 2.2.6) [84]. The full dataset includes 5 pairs of presumed first-degree relatives, 5 pairs of second-degree relatives, and 4 pairs of putative third-degree relatives (Additional file 1: Table S1).

To assess the impact of including close relatives in our selection scans, we randomly excluded one individual from each close-relative pair and re-estimated ZHp on the downsampled data. We then estimated the Pearson correlation between matched genomic regions in each downsampled and complete population. Data were plotted using the “ggpubr: ‘ggplot2’” package in R (version 0.4.0) [85].

We used two approaches to assess levels of genetic relatedness among populations. We first thinned SNPs to one variant per 1 kb interval for all samples using VCFtools (version 0.1.16) [82] and then projected the thinned data into two dimensions using a principal component analysis (Plink version 1.9) [86]. We also constructed a maximum likelihood phylogenetic tree from the 154 wild mouse genomes using PhyML (version 3.0) [87]. The best-fit nucleotide substitution model was determined using jModeltest (version 2.1.7) [88]. The resulting tree was visualized in MEGA (version 7) [89].

### Demographic estimation and the distribution of neutral diversity

The evolutionary history of the house mouse is a complex web of demographic processes, including migration and changes in population size. To distinguish regions of true positive selection from outliers of the neutral distribution of diversity, we derived the expected distribution of neutral diversity in each surveyed population. First, we used *angsd* (version 0.935) to calculate the site allele frequency likelihood based on individual genotype likelihoods, assuming that each population is in Hardy-Weinberg Equilibrium [90]. This output was then used to generate the site frequency spectrum across each population using *angsd* realSFS*.*

For each surveyed population, we then inferred population-specific demographic parameters using ∂a∂i [91]. Parameter estimation was performed from 1000 putatively neutral autosomal non-coding regions, each 300 kb in length. Assuming two generations per year and a mutation rate of 5.7 × 10^−9^ per bp [92], we ran a “one population two changes model” which assumes that the initial population split from an ancestral population, experienced a bottleneck, and subsequently expanded. The parameter estimation optimization procedure was repeated 10 times to ensure that maximum likelihood estimates were insensitive to different starting values and ranges.

Estimated demographic parameters from ∂a∂i were used to seed neutral population-specific coalescent simulations in *ms* [93]. A total of 10,000 independent simulations were performed for each population. The invoked commands for each population are: America (-t 783 -eN 0.037518545 2.946817641 -eN 0.056984026 0.048572057), France (-t 198 -eN 2.47046851 0.914897206 -eN 0.122115052 0.247412073), Germany (-t 620 -eN 0.216181765 1.537319741 -eN 0.175112317 0.13052885), Iran (-t 2449 -eN 0.13237575 0.039843163 -eN 0.08026151 0.152704587), India (-t 1230 -eN 0.368975226 0.406795355 -eN 0.233061222 0.640302075), Taiwan (-t 1017 -eN 0.00605714 0.069121519 -eN 0.005099528 0.024048706), Afghanistan (-t 878 -eN 0.140051542 0.07599747 -eN 0.073712335 0.320965943), Czech (-t 1229 -eN 0.06273194 0.124720872 -eN 0.124106941 0.051288075), Kazakhstan (-t 702 -eN 0.047809205 0.314506899 -eN 0.100143924 0.114369593), and *M. spretus* (-t 721 -eN 0.038447928 0.191823599 -eN 0.095940492 0.113808736). Additional file 3: Figure S2 shows that the simulated neutral diversity distribution broadly matches the observed distribution of diversity for each population.

### Identifying footprints of positive selection

As a beneficial allele increases in frequency under positive selection, it carries linked genetic variants with it, leaving behind a reduction in diversity at the targeted locus. To identify this signature of locally depressed diversity in the mouse genome, we computed three population genomic diversity statistics in 20 kb windows (10 kb sliding steps) across the genome: pool heterozygosity (*H*_p_) [33], nucleotide diversity (*π*) [34], and Tajima’s *D* [35]. Our analysis was restricted to variants on the autosomes.

Windows with < 50 SNPs were excluded, resulting in the elimination of ~ 0.3 to ~ 4% of all windows, depending on the population. Diversity statistics were normalized for each population to enable comparison across analyses. The significance threshold was obtained based on the extreme value from the coalescent simulation in a one-tailed direction of the selective sweep. Adjacent windows were then collapsed to form single candidate regions, similar to a previous study [94].

We focus on extreme regions in the observed *H*p distribution that are also supported by at least one of the other tested statistics: *π* or Tajima’s *D*. Although the computed statistics are not strictly independent of one another, they do encapsulate slightly different aspects of the patterning of genetic variation.

### Filtering for windows exhibiting non-diploid state

Read depth was computed in 1000 bp windows across each sequenced mouse genome using *mosdepth* [95]. Absolute read depth values were corrected for GC-content biases following established methods [96] and standardized by the genome-wide average read depth to convert to copy number (CN) estimates. We approximated all CN estimates to their nearest whole number (e.g., CN > 1.5 and CN < 2.5 correspond to CN = 2) and then retained only windows with CN = 2 in each sample. Next, we used the “—intersect” option of the bedops version 2.4.39 [97] to retain only windows where CN = 2 for all the analyzed samples. Finally, we used these CN metrics to filter and discard positive selection regions carrying a non-diploid copy number using the “intersect” option of bedtools version 2.29.2 [98]

### Association with Mendelian traits and functional classification of putative sweep genes

We estimated the fraction of candidate sweep genes that overlap with genes in the OMIM database (https://www.omim.org/, retrieved October 22, 2020; Additional file 16: Table S6) and GWAS catalog (https://www.ebi.ac.uk/gwas/, accessed March 6, 2021). We then compared this fraction to the genome-wide null expectation using a simulation procedure. Briefly, we randomly selected a set of non-overlapping genomic regions size-matched to the distribution of the observed sweep windows. We then identified genes within the simulated windows and computed the fraction of simulated regions that overlap with entries in the OMIM and GWAS databases. We repeated this simulation procedure 1000 times to derive the expected frequency of both OMIM and GWAS genes in sweep windows.

For functional classification, we retrieved genes within each candidate selective sweep region using Ensembl BioMart version 102 [99]. These gene lists were used for GO and KEGG analyses using the Database for Annotation, Visualization, and Integrated Discovery (DAVID version 6.8) [100]. We used all RefSeq genes in the *M. musculus* genome as background. Overrepresented gene clusters were identified by Fisher’s exact tests (*p* < 0.05) and visualized in ggplot2 [85].

### Gene expression analyses

Publicly available transcriptome sequencing reads from 10 different tissues (gut, brain, heart, liver, lung, spleen, kidney, testis, thyroid, muscle) were obtained from wild-caught *M. m. domesticus* mice from Iran, France, and Germany [17]. Mapped reads were compiled into a count matrix using the “featureCounts” command in the Rsubread package (version 2.6.4). The resulting count matrix was then used to run a differential gene expression analysis across populations with the *edgeR* [101] and *DESeq2* [102] pipelines. The threshold for significance was set at *p* < 0.01 in *edgeR* and adjP < 0.05 in *DESeq2*. Both methods produced largely overlapping sets of significantly differentially expressed genes across the populations. The resulting data from the *DESeq2* was further analyzed.

We performed simulation analysis to assess the significance of the overlap between genes under selection and differentially expressed genes. Simulations were independently executed for each of the 10 surveyed tissues. Briefly, for a given tissue, we randomly sampled the number of genes under positive selection from the full set of gene expression measures. This subsampling procedure was repeated 10,000 times. For each simulated dataset, we then calculated the fraction of randomly sampled genes that are significantly differentially expressed across populations. An empirical *p* value was calculated by determining the proportion of times the simulated overlap was greater than the true overlap between selection genes and differentially expressed genes (Additional file 7: Table S2).

## Supplementary Information

**Additional file 1: Table S1.** Relatedness among samples.

**Additional file 2: Figure S1.** Pearson correlation coefficient for ZHp between the down-sampled data and the analysis on all samples.

**Additional file 3: Figure S2.** The distribution of simulated neutral and the observed diversity in each population.

**Additional file 4: Figure S3.** Genome-wide distribution of positive selection signals in four populations of *M. m. domesticus.* The horizontal lines correspond to the significance threshold for defining windows under positive selection.

**Additional file 5: Figure S4.** Genome-wide distribution of positive selection signals in two populations of *M. m. castaneus* and in *M. spretus.* The horizontal lines correspond to the significance threshold for defining windows under positive selection.

**Additional file 6: Figure S5.** Genome-wide distribution of positive selection signals in three populations of *M. m. musculus.* The horizontal lines correspond to the significance threshold for defining windows under positive selection.

**Additional file 7: Table S2.** Candidate selective sweep regions, gene expression, GO, and KEGG analyses in the four populations of *M. m. domesticus.*

**Additional file 8: Table S3.** Candidate selective sweep regions, GO, and KEGG analyses in the two populations of *M. m. castaneus.*

**Additional file 9: Table S4.** Candidate selective sweep regions, GO, and KEGG analyses in the three populations of *M. m. musculus.*

**Additional file 10: Table S5.** Candidate selective sweep regions, GO, and KEGG analyses in *M. spretus.*

**Additional file 11: Figure S6.** Cryptic structural variation at the *Skint* gene cluster (chr4:112.08–112.60 Mb) yields signals consistent with a selective sweep in the Indian *M. m. castaneus*. (**a**) ZHp for the two *M. m. castaneus* populations. (**b**) An Indian diploid sample for this locus. (**c**) The deletion haplotype is at 90% frequency in the Indian population. Panel (**d**) presents the organization of the *Skint* paralogs across this region.

**Additional file 12: Figure S7.** Pathway and functional overrepresentation (*p*<0.05) of putative signals of positive selection in *M. m. domesticus*.

**Additional file 13: Figure S8.** Pathway and functional overrepresentation (*p*<0.05) of putative signals of positive selection in *M. m. castaneus.*

**Additional file 14: Figure S9.** Pathway and functional overrepresentation (*p*<0.05) of putative signals of positive selection in *M. m. musculus*.

**Additional file 15: Figure S10.** Pathway and functional overrepresentation (*p*<0.05) of putative signals of positive selection in *M. spretus*.

**Additional file 16: Table S6.** Candidate selective sweep genes and their association with Mendelian and complex traits*.*

**Additional file 17: Figure S11.** RNA expression levels of *Amy1*, *Cry1*, *Epas1*, and *Mgam* in various tissues (A-J) collected from *M. m. domesticus* populations of Germany (GR), Iran (IR), and France (FR). RNA expression level is represented by log normalized counts of reads (y-axis) in the populations (x-axis). Genes highlighted in red have significant (Likelihood ratio test, adjP < 0.05) differential gene expression across populations in the particular tissue.

**Additional file 18: Figure S12.** Copy number architecture across the amylase locus in *M. m. domesticus* populations. “Sweep” is the locus experiencing positive selection, “SV” corresponds to a region of structural variation, and “mm10 gap” labels a gap in the mm10 reference genome.

**Additional file 19: Figure S13.** The distribution of average quality scores and read depth across samples.

## Data Availability

All the genome sequences used in this study were obtained from three previously published studies [6, 17, 18].
