# Supplementary figures and images for: Selection shapes the landscape of functional variation in wild house mice
Source: BMC Biol. 2021 Nov 19;19:239. doi: 10.1186/s12915-021-01165-3 (PMC8603481; doi:10.1186/s12915-021-01165-3)

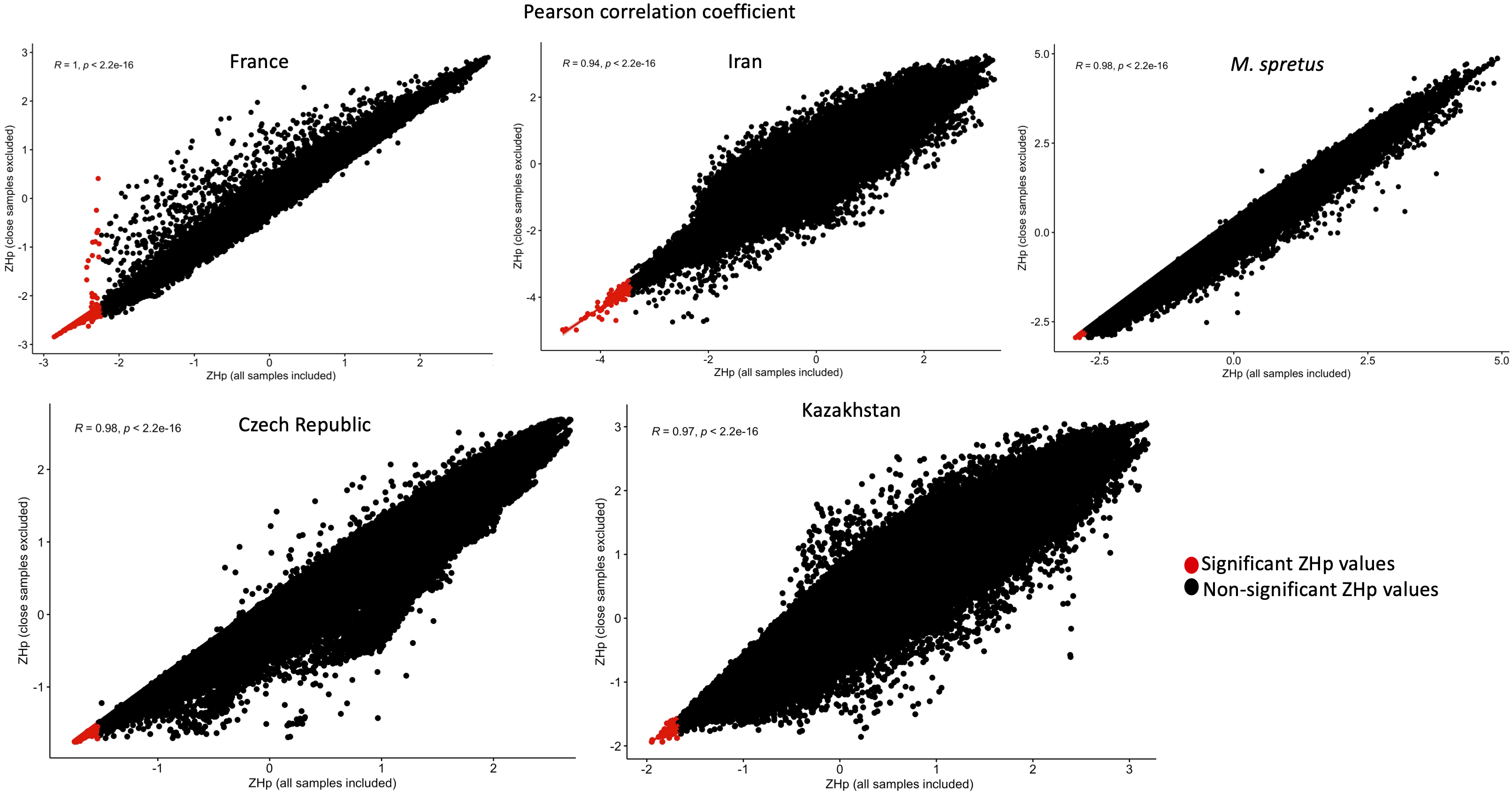

Supplement: Supplementary file 2 — Additional file 2: Figure S1. Pearson correlation coefficient for ZHp between the down-sampled data and the analysis on all samples. [file 12915_2021_1165_MOESM2_ESM.tiff]

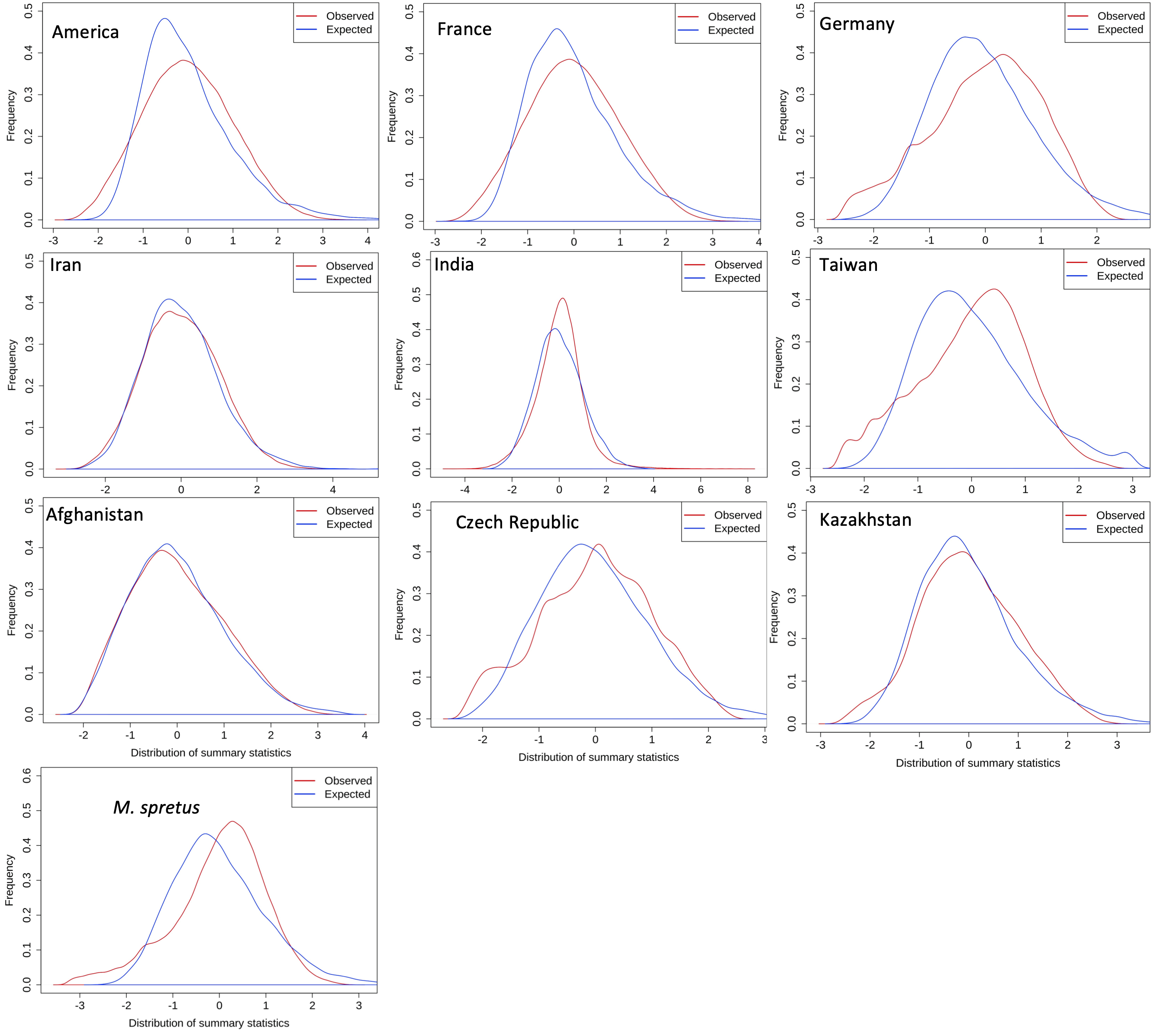

Supplement: Supplementary file 3 — Additional file 3: Figure S2. The distribution of simulated neutral and the observed diversity in each population. [file 12915_2021_1165_MOESM3_ESM.tiff]

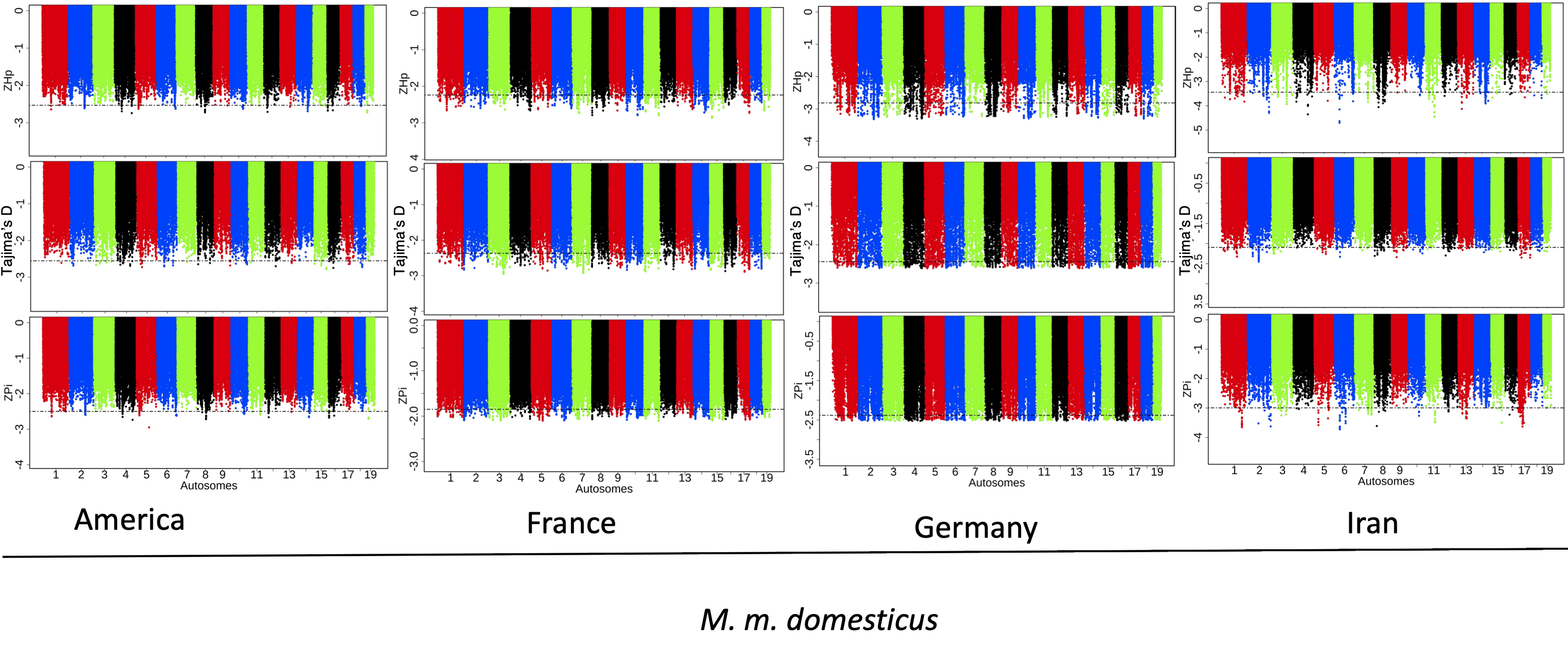

Supplement: Supplementary file 4 — Additional file 4: Figure S3. Genome-wide distribution of positive selection signals in four populations of M. m. domesticus. The horizontal lines correspond to the significance threshold for defining windows under positive selection. [file 12915_2021_1165_MOESM4_ESM.tiff]

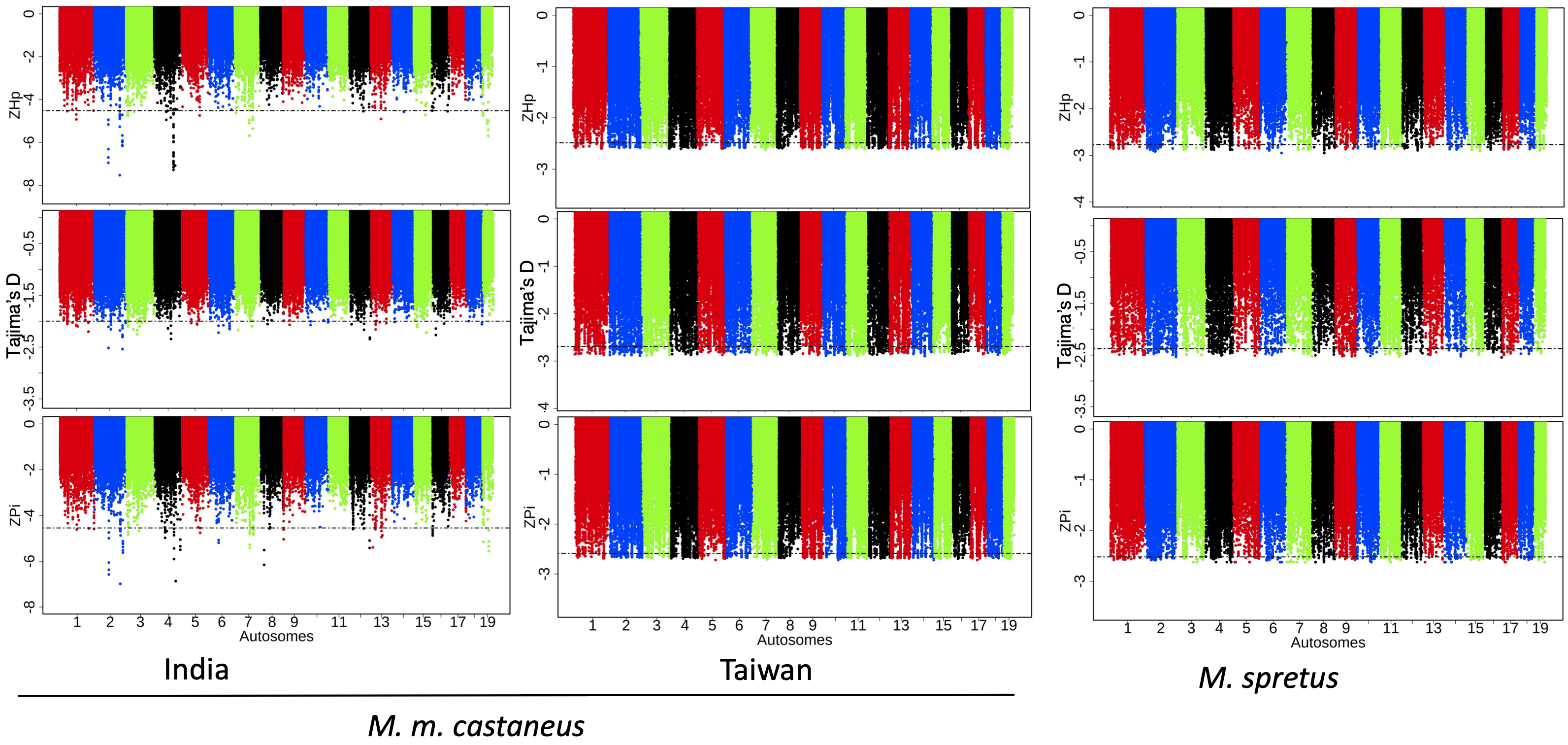

Supplement: Supplementary file 5 — Additional file 5: Figure S4. Genome-wide distribution of positive selection signals in two populations of M. m. castaneus and in M. spretus. The horizontal lines correspond to the significance threshold for defining windows under positive selection. [file 12915_2021_1165_MOESM5_ESM.tiff]

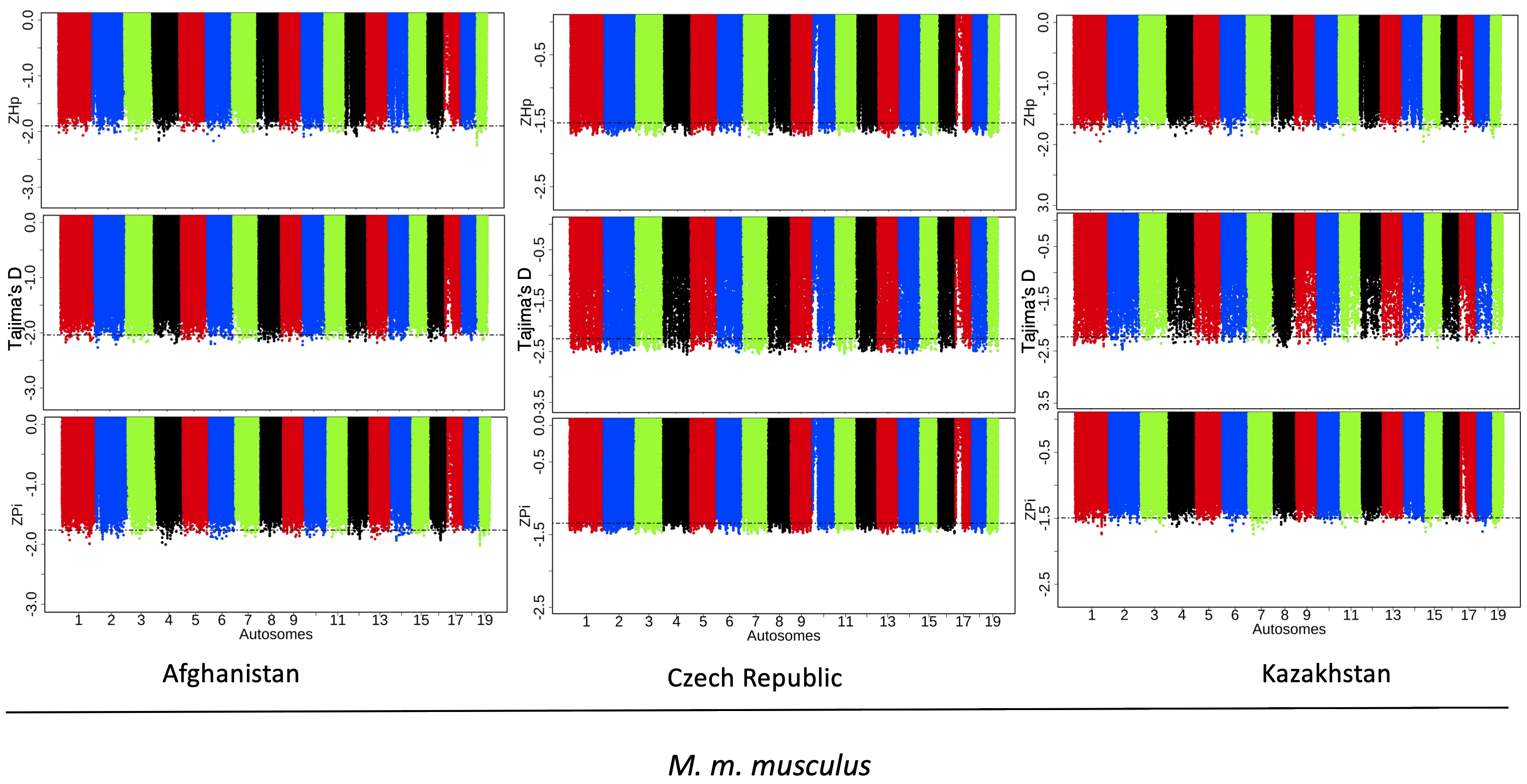

Supplement: Supplementary file 6 — Additional file 6: Figure S5. Genome-wide distribution of positive selection signals in three populations of M. m. musculus. The horizontal lines correspond to the significance threshold for defining windows under positive selection. [file 12915_2021_1165_MOESM6_ESM.tiff]

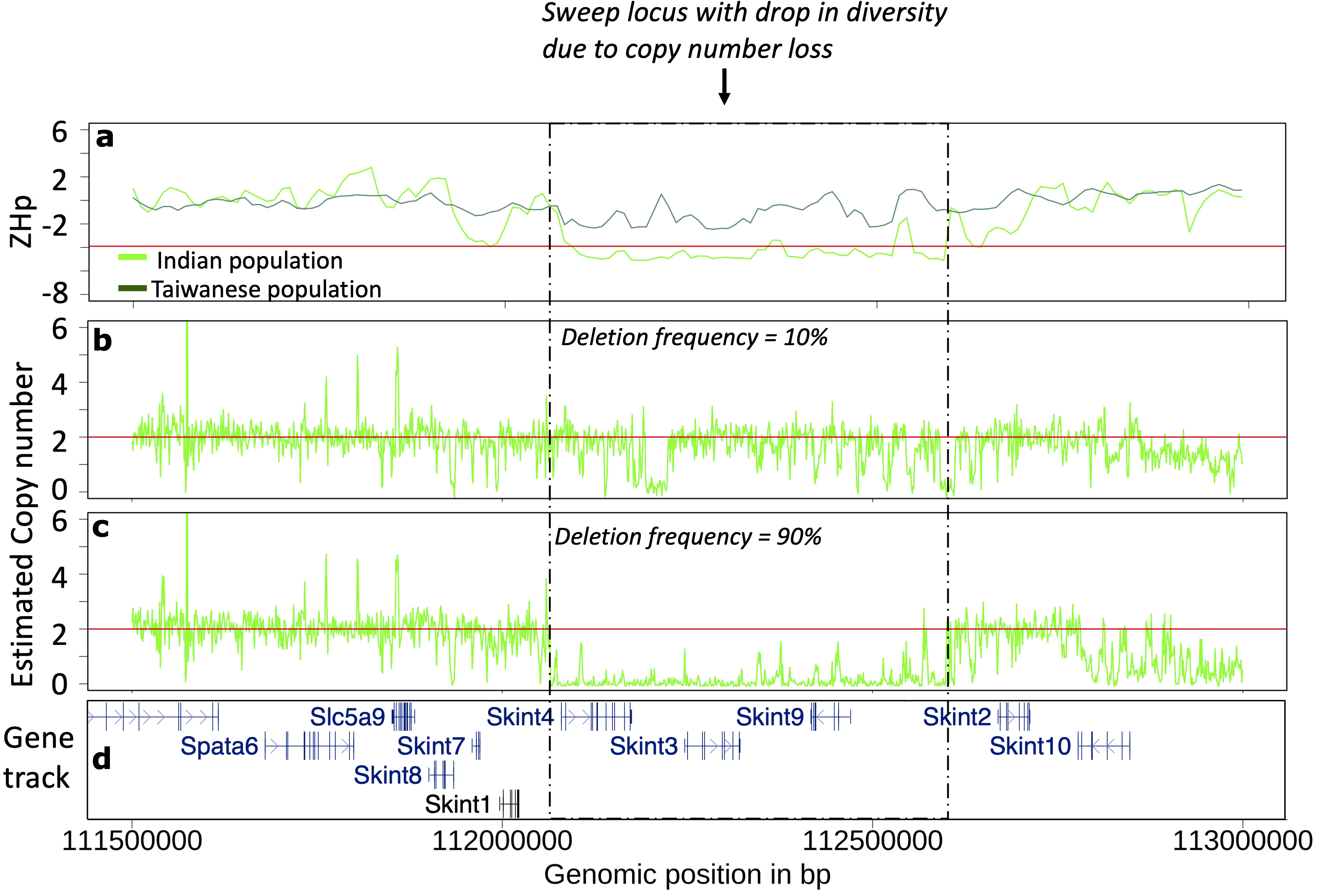

Supplement: Supplementary file 11 — Additional file 11: Figure S6. Cryptic structural variation at the Skint gene cluster (chr4:112.08–112.60 Mb) yields signals consistent with a selective sweep in the Indian M. m. castaneus. (a) ZHp for the two M. m. castaneus populations. (b) An Indian diploid sample for this locus. (c) The deletion haplotype is at 90% frequency in the Indian population. Panel (d) presents the organization of the Skint paralogs across this region. [file 12915_2021_1165_MOESM11_ESM.tiff]

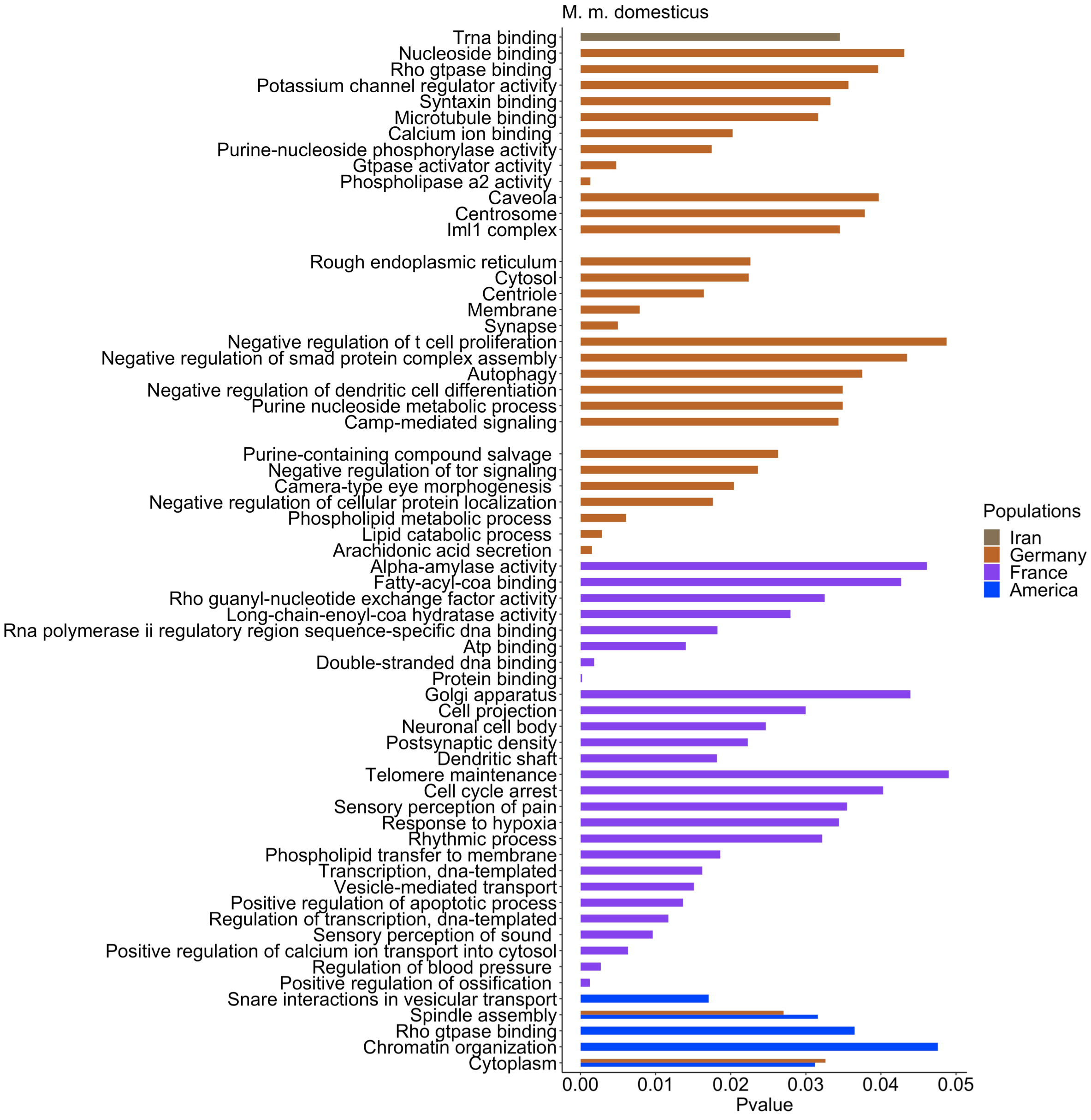

Supplement: Supplementary file 12 — Additional file 12: Figure S7. Pathway and functional overrepresentation (p<0.05) of putative signals of positive selection in M. m. domesticus. [file 12915_2021_1165_MOESM12_ESM.tiff]

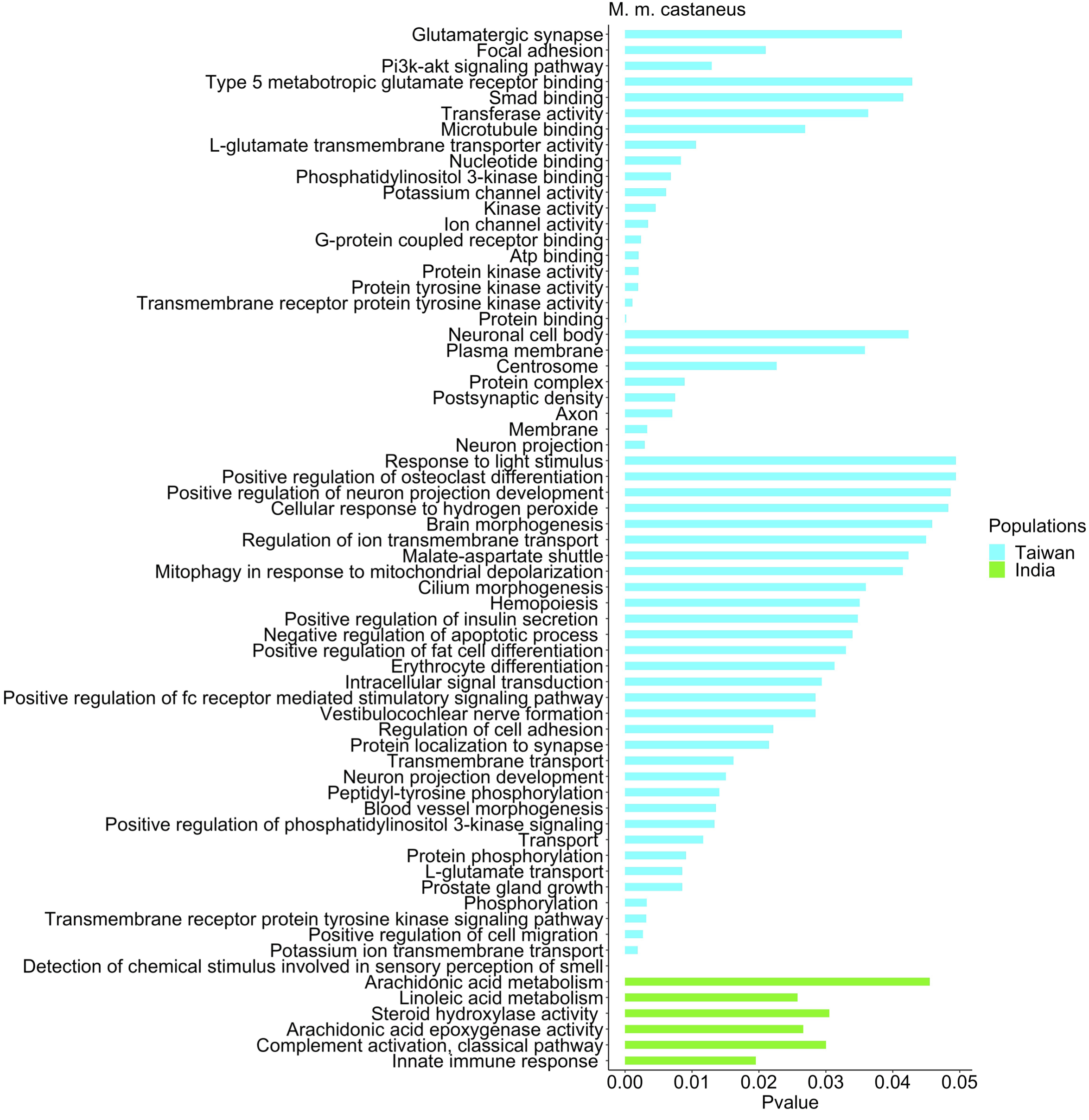

Supplement: Supplementary file 13 — Additional file 13: Figure S8. Pathway and functional overrepresentation (p<0.05) of putative signals of positive selection in M. m. castaneus. [file 12915_2021_1165_MOESM13_ESM.tiff]

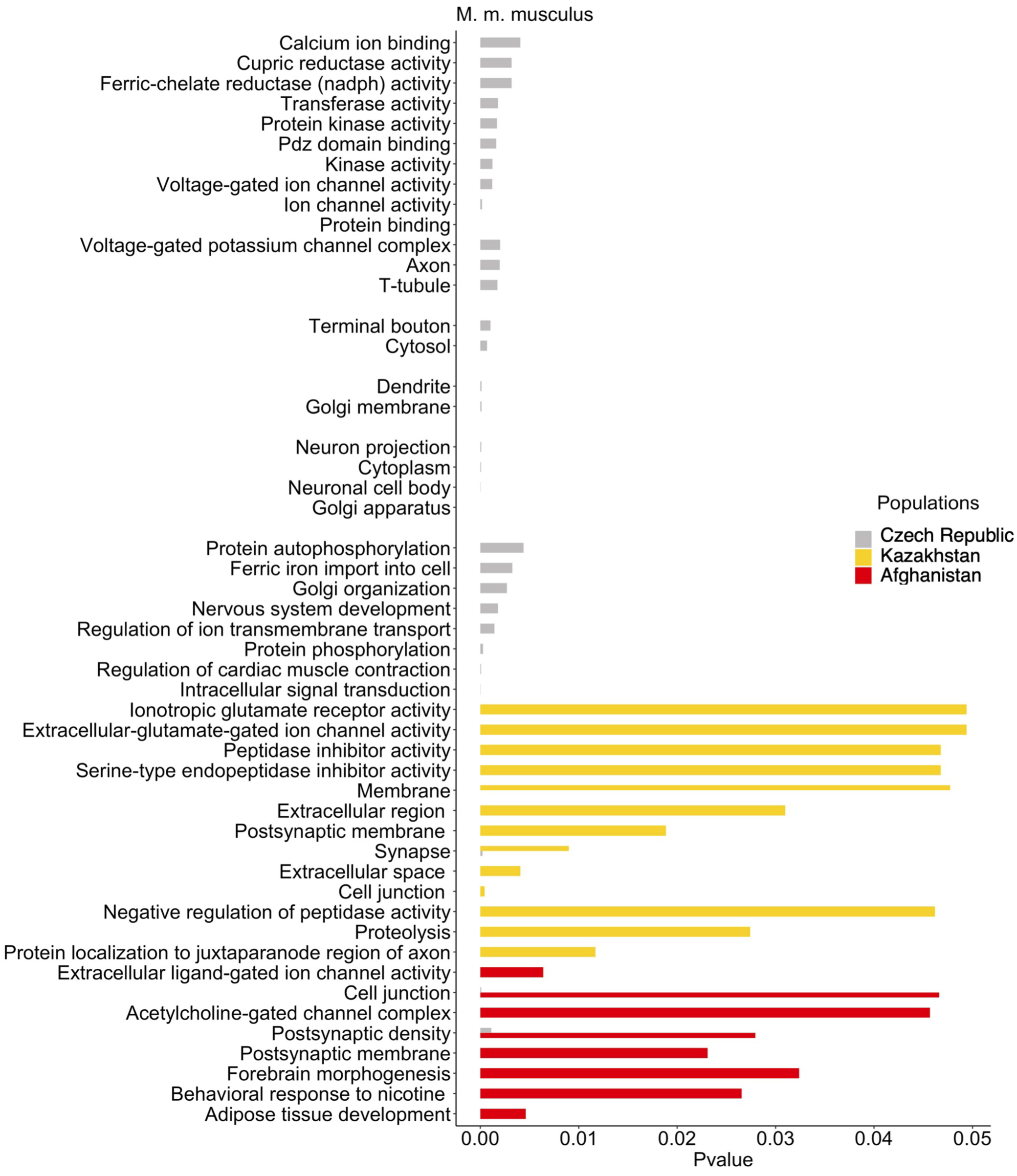

Supplement: Supplementary file 14 — Additional file 14: Figure S9. Pathway and functional overrepresentation (p<0.05) of putative signals of positive selection in M. m. musculus. [file 12915_2021_1165_MOESM14_ESM.tiff]

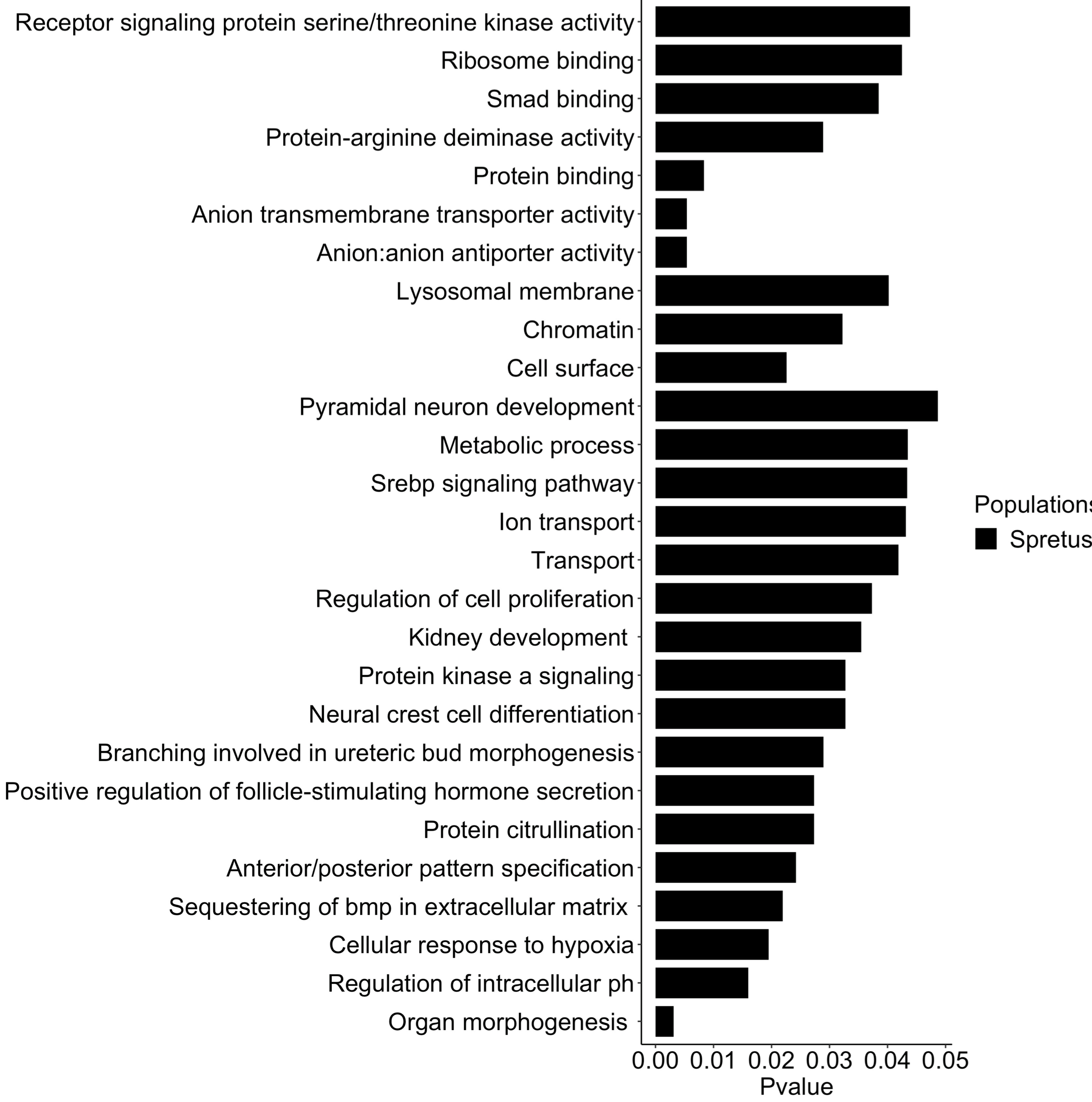

Supplement: Supplementary file 15 — Additional file 15: Figure S10. Pathway and functional overrepresentation (p<0.05) of putative signals of positive selection in M. spretus. [file 12915_2021_1165_MOESM15_ESM.tiff]

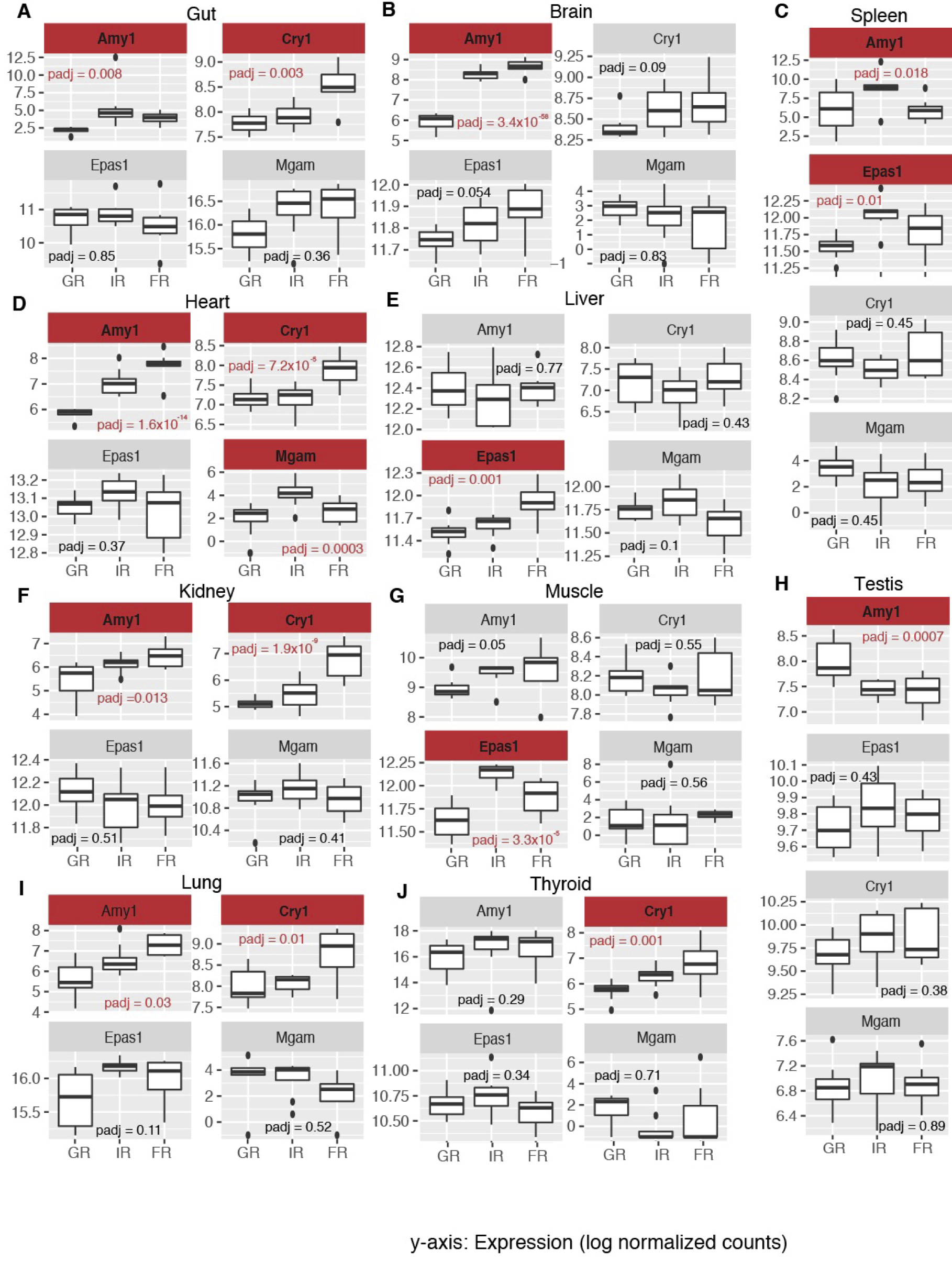

Supplement: Supplementary file 17 — Additional file 17: Figure S11. RNA expression levels of Amy1, Cry1, Epas1, and Mgam in various tissues (A-J) collected from M. m. domesticus populations of Germany (GR), Iran (IR), and France (FR). RNA expression level is represented by log normalized counts of reads (y-axis) in the populations (x-axis). Genes highlighted in red have significant (Likelihood ratio test, adjP < 0.05) differential gene expression across populations in the particular tissue. [file 12915_2021_1165_MOESM17_ESM.tiff]

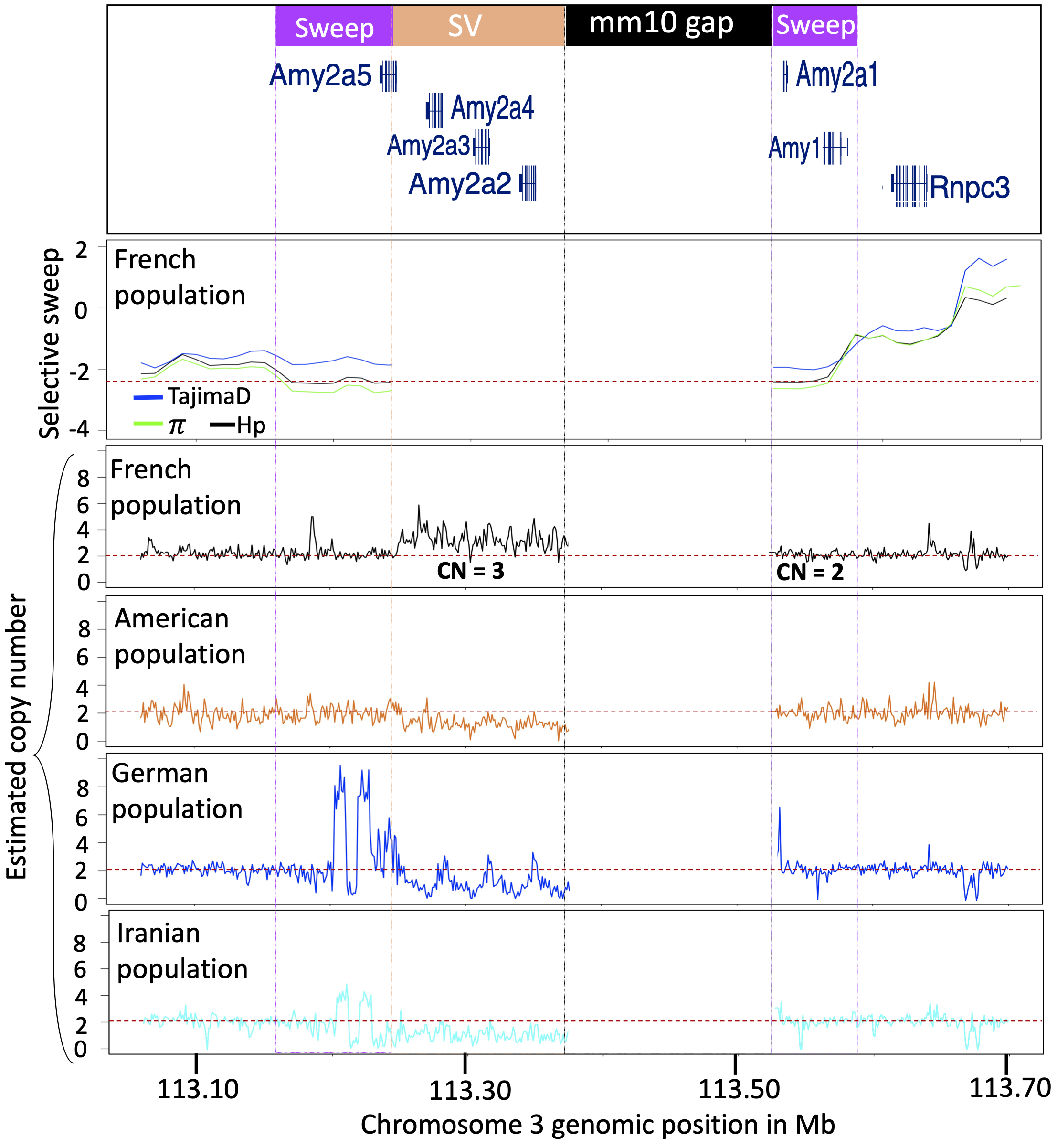

Supplement: Supplementary file 18 — Additional file 18: Figure S12. Copy number architecture across the amylase locus in M. m. domesticus populations. “Sweep” is the locus experiencing positive selection, “SV” corresponds to a region of structural variation, and “mm10 gap” labels a gap in the mm10 reference genome. [file 12915_2021_1165_MOESM18_ESM.tiff]

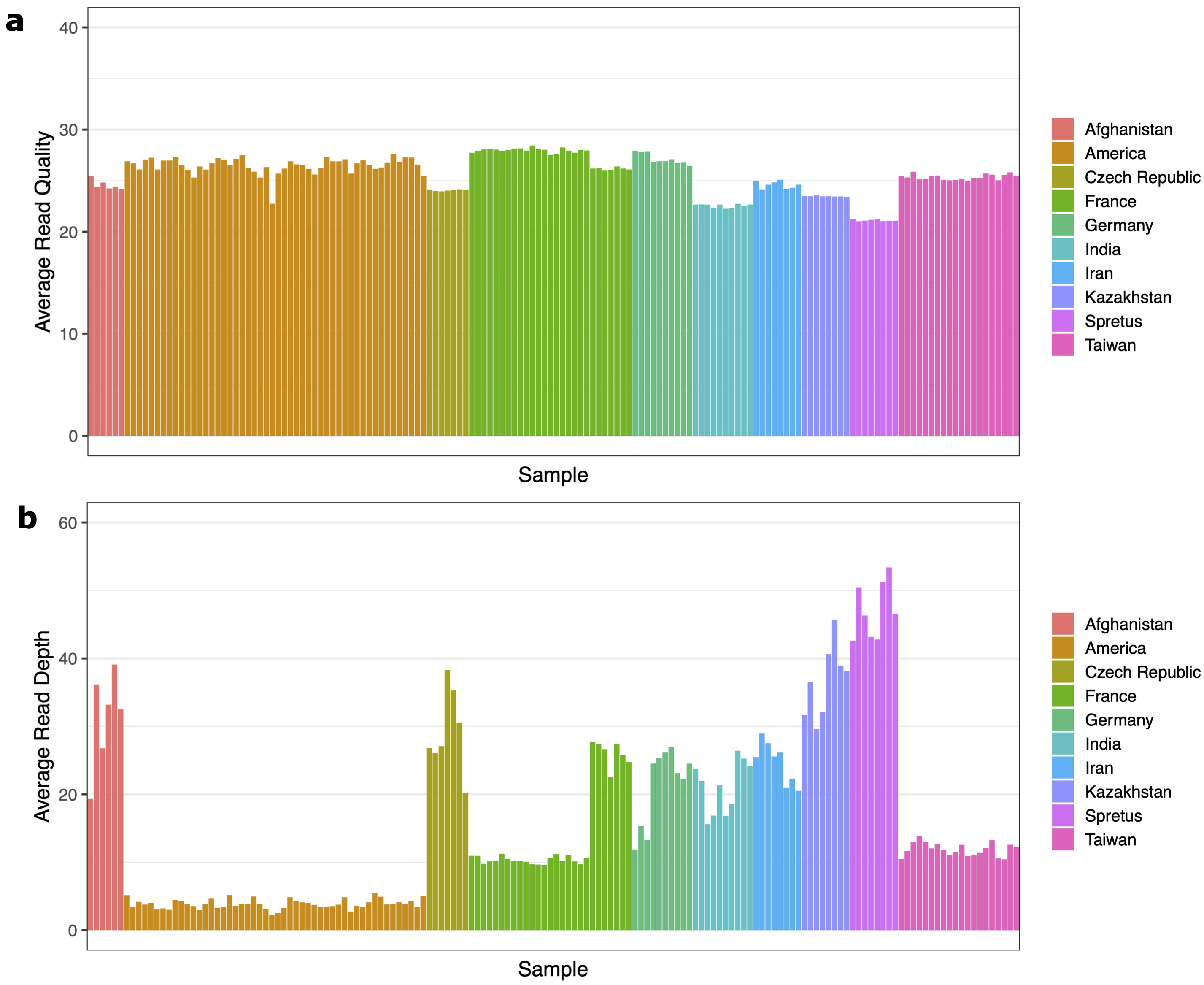

Supplement: Supplementary file 19 — Additional file 19: Figure S13. The distribution of average quality scores and read depth across samples. [file 12915_2021_1165_MOESM19_ESM.tiff]
